# Gut Microbiota‐Associated Metabolites Affected the Susceptibility to Heart Health Abnormality in Young Migrants at High‐Altitude: Gut Microbiota and Associated Metabolites Impart Heart Health in Plateau

**DOI:** 10.1002/EXP.20240332

**Published:** 2025-06-12

**Authors:** Yongqiang Zhou, Zhexin Ni, Jingjing Liu, Dezhi Sun, Pan Shen, Xi Chen, Gaofu Li, Zhijie Bai, Yangyi Hu, Ningning Wang, Rui Wang, Lina Guan, Yihao Wang, Xianglin Tang, Yungang Lu, Baokun He, Haitao Lu, Wei Zhou, Yue Gao

**Affiliations:** ^1^ Beijing Institute of Radiation Medicine Beijing China; ^2^ School of Chinese Medicine Hong Kong Traditional Chinese Medicine Phenome Research Center State Key Laboratory of Environmental and Biological Analysis Hong Kong Baptist University Hong Kong China; ^3^ Key Laboratory of Systems Biomedicine (Ministry of Education) Shanghai Center for Systems Biomedicine Shanghai Jiao Tong University Shanghai China; ^4^ Department of Respiratory Medicine Army 954 Hospital Shannan, Tibet China; ^5^ General Hospital of Xinjiang Military Command Urumqi Xinjiang China; ^6^ Department of Genetics The University of Texas MD Anderson Cancer Center Houston Texas USA; ^7^ Department of Gastroenterology Shanghai General Hospital Shanghai Jiao Tong University School of Medicine Shanghai China; ^8^ Shanghai Institute of Hematology State Key Laboratory of Medical Genomics National Research Center for Translational Medicine at Shanghai Ruijin Hospital Shanghai Jiao Tong University School of Medicine Shanghai China; ^9^ State Key Laboratory of Kidney Diseases Chinese PLA General Hospital Beijing China

**Keywords:** glycolysis, heart health abnormality, metabolomic, metagenomic, plateau migrants

## Abstract

Young migrants, particularly those at high altitudes, are predisposed to heart health abnormalities, including high‐altitude heart disease. Despite the profound impact of hypobaric hypoxia on the gut microbial community, the understanding of the roles played by gut microbiota and gut microbiota‐associated serum metabolites in high‐altitude heart diseases remains limited. Therefore, we conducted a comprehensive multi‐omics analysis involving 230 graduates from the same university, with 163 Tibetan Plateau migrants and 67 Chengdu Plain residents, and identified 206 differential metabolites (82 in serum and 124 in feces) and 369 species that differed between migrants and residents. Among these, 27 microbial species and four metabolites (Ketoglutaric acid, L‐Aspartic acid, 3‐Guanidinopropionic acid, betaine) detected in both serum and feces were found to be associated with migrants exhibiting compromised heart health, as diagnosed through clinical examinations. Notably, the abundances of *Veillonella rogosae* and *Streptococcus rubneri* were correlated with serum levels of L‐Aspartic acid, betaine, and Ketoglutaric acid in heart health‐abnormal individuals. Validation of these microbiome biomarkers and gut microbiota‐associated serum metabolites in an independent cohort demonstrated their excellent predictive ability for indicating heart health abnormalities in migrants (AUC = 0.7857). Furthermore, supplementation with these identified species or gut microbiota‐associated serum metabolites effectively mitigated hypobaric hypoxia‐induced increases in serum lactate, glycolysis, myocardial damage, and cardiac hypertrophy. Integrated analysis revealed that the alterations in the gut microbiome negatively regulated key metabolic pathways such as the malate‐aspartate shuttle, tricarboxylic acid cycle, and oxidative phosphorylation in heart health‐abnormal individuals. The migration to high‐altitude plateaus significantly reshaped the gut microbiome and metabolome signatures. Lower abundances of *Veillonella rogosae*, *Streptococcus rubneri*, and gut microbiota‐associated serum metabolites promoted the remodeling of metabolic processes, thereby increasing susceptibility to high‐altitude heart health abnormalities. Overall, our findings elucidate the microbial mechanisms underlying high‐altitude heart disease and provide valuable insights for potential early intervention strategies in this context.

## Graphical abstract

Long‐term migration to high‐altitude areas can readily result in chronic high‐altitude diseases; however, effective prevention and treatment methods are currently lacking. This study employed a multi‐omics approach, integrating metagenomics and metabolomics, to discover and validate that the decreased abundance of *Veillonella rogosae*, *Streptococcus rubneri*, and gut microbiota‐associated serum metabolites, contributes to increased susceptibility to high‐altitude heart abnormalities across multiple dimensions, including population studies, animal models, and cellular experiments. These findings enhance the early diagnosis of high‐altitude heart abnormalities and provide potential therapeutic interventions for high‐altitude heart disease.

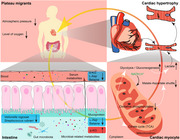

**Abbreviations**
2‐DG: 2‐deoxy‐D‐glucose
3‐GUA: 3‐guanidinopropionic acid
AMS: Acute mountain sickness
ANOSIM: Analysis of similarities
BMI: Body mass index
CKMB: Creatine kinase isoenzymes
CMS: Chronic mountain sickness
CTNI: Cardiac troponin I
CVD: Cardiovascular disease
ECAR: Extracellular acidification rate
ECG: Electrocardiogram
EF%: Ejection fraction
ELISA: Enzyme‐linked immunosorbent assay‐sandwich technique
F/B: Firmicutes to Bacteroides
FCG: Frequency‐domain cardiogram
FS%: Fractional shortening
GMSMs: Gut microbiota‐associated serum metabolites
HA: High‐altitude group
HAHD: High‐altitude heart disease
HBDH: Hydroxybutyrate dehydrogenase
HE: Hematoxylin‐eosin
HH: Hypobaric hypoxia group
HH‐A: Heart health‐abnormal group
HH‐N: Heart health‐normal group
HIF: Hypoxia‐inducible factor
IHD: Ischemic heart disease
KEGG: Kyoto Encyclopedia of Genes and Genomes
L‐Asp: L‐Aspartic
LDH: Lactate dehydrogenase
Lefse: Linear discriminant analysis effect size
MA: Malate‐aspartate
MSE: Mean square error
NA: Normal atmosphere group
OD: Absorbance
ORFs: Open reading frames
PAH: Pulmonary arterial hypertension
PCA: Principal component analysis
PERMANOVA: Permutational multivariate analysis of variance
PL: Plain group
PLS‐DA: Partial least squares discriminant analysis
PSM: Propensity score matching
RF: Random forests
ROC: Receiver operating characteristic curve
Rot/AA: Mitochondrial electron transport chain
*S. rubneri*
: *Streptococcus rubneri*
SCFAs: Short‐chain fatty acids
SD: Standard deviation
STAMP: Statistical analysis of metagenomic profiles
TCA: Tricarboxylic acid cycle
TMAO: Trimethylamine oxide
UCG: Ultrasonic cardiogram
*V. rogosae*
: *Veillonella rogosae*
α‐KG: Ketoglutaric acid

## 1 Introduction

The gut microbiome that inhabits the human intestinal tract is a complex community of symbiotic microorganisms, consisting of bacteria, viruses, fungi, and other protozoans [1]. In recent years, the gut microbiota has gained recognition for its reciprocal commensal relationship with humans and its role as a driver of the host's physiological activities. For instance, the gut microbiota actively participates in the generation and utilization of essential nutrients, such as vitamins, essential amino acids, and short‐chain fatty acids (SCFAs), facilitated by microbiome‐specific enzymes involved in food fermentation and digestion [2]. Furthermore, the major phyla of the gut microbiota not only impact bile acid synthesis and metabolism but also contribute to the maintenance of immune homeostasis and defense against pathogen invasion [3]. Conversely, alterations in the composition and function of these diverse intestinal bacterial communities, closely linked to lifestyle and environmental factors, significantly contribute to the development of various diseases, including metabolic syndrome, cardiovascular disease (CVD), circadian dysrhythmia, and even cancer [4]. Therefore, the temporal stability and diversity of the gut microbiota are crucial elements in maintaining individual health.

Exposure to high‐altitude environments has been shown to induce changes in the structure of gastrointestinal microbiota. Several human studies have documented significant alterations in gut microbiome diversity and/or abundance in both visitors and migrants at high altitudes [5]. Generally, there is a decrease in total aerobes accompanied by an increase in obligate and facultative anaerobes in the gut microbiota composition among individuals transitioning from plain to plateau. Following prolonged residence at high altitudes, the characteristics of the microbial consortium in migrants gradually align with those of the native plateau inhabitants. Interestingly, an increase in the relative abundance of *Prevotella* has been correlated with the onset of acute mountain sickness (AMS) during high‐altitude acclimatization [6]. Furthermore, differences in the gut microbiome characterized by a high ratio of *Firmicutes* to *Bacteroides* (*F/B*) and a significant decrease in *Prevotella*, both of which influence responses to a high‐fat diet, have also been observed in Chinese Han individuals residing in Tibet for more than four years [5, 6, 7]. These findings underscore the pivotal role of gut microbiome changes in host responses to high‐altitude environments. However, a more comprehensive characterization of the gut microbiome signature, particularly disease‐specific profiles, in individuals migrating to high‐altitude regions is warranted due to limited sample sizes and other confounding factors in previous studies.

High‐altitude heart disease (HAHD), a form of chronic mountain sickness (CMS) characterized by pulmonary arterial hypertension (PAH) and right heart hypertrophy, often arises from maladaptation and dysfunction of the cardiovascular system at high altitudes [8]. In addition to PAH, the myocardium's susceptibility to hypobaric hypoxia, the primary environmental stressor on the plateau encountered by all high‐altitude residents, contributes to the development of altitude‐related cardiac hypertrophy and potentially congestive heart failure [9]. At a mechanistic level, intricate cellular responses are triggered to orchestrate cardiac adaptation to hypoxia primarily through the hypoxia‐inducible factor (HIF) signals in response to ambient hypoxia [10]. Moreover, HIF signals activated in the intestine have been shown to play a role in maintaining intestinal commensalism homeostasis and producing microbial metabolites, both crucial for safeguarding cardiomyocytes against hypoxic and/or ischemic damage [11]. Recent findings and animal studies have underscored that significant changes in the body's external environment can induce alterations in the gut microbiome and microbial metabolites, leading to myocardial injury, compromised myocardial perfusion, and increased susceptibility to ischemic heart disease (IHD) and myocardial hypertrophy triggered by low‐pressure hypoxia. However, human studies elucidating the microbiome and metabolome characteristics of HAHD in high‐altitude migrants, particularly during the early stages before clinical manifestation, are lacking [12, 13]. Therefore, this study aimed to investigate the role of the gut microbiome in HAHD development and its underlying mechanisms by profiling the gut microbiome spectrum and plasma and microbial metabolome signatures in three distinct groups: healthy individuals at low altitudes, healthy individuals at high altitudes, and individuals at high altitudes with a potential risk of heart health abnormalities. Subsequently, an integrated analysis of the gut microbiome and metabolome from humans and gut microbiota, supplemented by experimental validation through animal studies, revealed that the hypobaric hypoxia environment led to a reduction in the abundance of *Veillonella rogosae* (*V.rogosae*), *Streptococcus rubneri* (*S. rubneri*), and related gut microbiota‐associated serum metabolites (GMSMs), shifting the energy metabolism of the gut and heart toward glycolysis and increasing susceptibility to heart health abnormalities at high altitudes. This comprehensive investigation provides insights into the microbiome and metabolome pathophysiological signatures associated with high‐altitude heart health abnormalities, which could be crucial for the prevention and intervention of HAHD.

## 2 Materials and Methods

### 2.1 Cohort Description

The study enrolled two distinct groups: Plain group (PL) comprising 105 adult males from the Chengdu plain in Sichuan, and high‐altitude group (HA) comprising 641 adult males from Tibet residing at altitudes ranging from 3500 to 4500 m. All participants completed a comprehensive basic information questionnaire for subsequent population screening. To ensure that the identified heart problems and alterations in gut microbiota were primarily influenced by environmental factors, precise inclusion and exclusion criteria were implemented [14]. To assure consistency in the origin of the population, we required that the plateau migrants resided in roughly similar geographic areas as the plains populations before migrating to the plateau. After applying these criteria, 67 individuals were selected for PL, and 163 individuals were chosen for HA, all of whom graduated from Chengdu Medical College. These two groups accepted detailed examinations, including electrocardiogram (ECG), ultrasonic cardiogram (UCG), and frequency‐domain cardiogram (FCG), conducted by the Army 954 Hospital. Based on the results of ECG, FCG, and UCG, individuals in HA were divided into two subgroups, including heart health‐normal group (HH‐N) (*n* = 42) and heart health‐abnormal group (HH‐A) (*n* = 35) by matching age, altitude, dietary habits, duration of migration to the plateau, and body mass index (BMI). To further validate the identified features, 20 volunteers with myocardial ischemia were recruited to form the abnormal group, while 21 volunteers were selected to form the normal group by matching age, altitude, dietary habits, duration of migration to plateau, and BMI.

The study received approval from the Ethics Committee of the Beijing Institute of Radiation Medicine, with the approval number AF/SC‐08/02.153. According to the Helsinki Declaration, all participants read and voluntarily completed the informed consent form prior to participating in the study.

### 2.2 Metagenomic Sequencing

Microbial DNA was extracted from stool samples following the manufacturer's protocols using the stool DNA Kit (Omega Bio‐tek, Norcross, GA, USA). Each sample underwent shearing of 1 µg genomic DNA by the Covaris S220 Focused‐ultrasonicator (Woburn, MA, USA), and sequencing libraries were prepared by Shanghai Biozeron Biological Technology Co. Ltd with a target fragment length of approximately 450 bp. Subsequently, all samples were sequenced on the Illumina HiSeq X platform in pair‐end 150 bp (PE150) mode. Raw sequence reads were subjected to quality trimming using Trimmomatic and quality control mapping against the human genome (version: hg19) using the BWA mem algorithm to eliminate adaptor contaminants and low‐quality reads [15]. For taxonomic annotation of the clean reads, Kraken2 was employed to classify the reads at seven phylogenetic levels using a customized Kraken database encompassing various microorganisms [16]. The abundance of taxonomy was estimated using Bracken, providing accurate species‐ and genus‐level abundance information. Furthermore, the clean sequence reads from each sample were assembled into contigs using MegaHit [17], and open reading frames (ORFs) were predicted with Prodigal [18]. The ORFs were clustered using CD‐HIT to generate a set of unique genes, with gene abundance calculated using salmon software [19, 20]. Functional annotations of the genes were obtained by searching against the Kyoto Encyclopedia of Genes and Genomes (KEGG) databases using BLASTX [21]. The specific functions and pathways of each sample were determined based on the KEGG pathway analysis of the annotated genes.

### 2.3 Metabolome Profiling of Serum/Fecal Samples

The serum samples were mixed with four‐fold volumes of iced acetonitrile, which included 0.001 mg/mL 4‐chloro‐DL‐phenylalanine as an internal standard. The mixture was incubated on ice for 15 min to facilitate protein removal. Following centrifugation at 20,000 g at 4°C for 10 min, the supernatants were carefully collected into new microcentrifuge tubes. After another round of centrifugation under the same conditions, the samples were transferred to sample vials for metabolomics analysis.

For fecal samples, 60 mg of the sample was mixed with 600 µL of 80% iced methanol, which included 0.001 mg/mL 4‐chloro‐DL‐phenylalanine as an internal standard. Zirconia crushing beads were added to the mixture, and the samples were disrupted using a vibratory crusher operating at 60 Hz. Subsequently, the samples were processed by ultrasonication in an ice bath for 10 min and left to stand at −20°C for 30 min. The supernatants were collected by centrifugation at 20,000 g under 4°C for 10 min and then mixed with three‐fold volumes of distilled water. After ultrasonication for 3 min to ensure even mixing, the samples were placed in a −20°C environment for 2 h. Finally, the supernatants obtained from centrifugation were transferred to sample vials for metabolomics analysis [22].

The metabolomes were analyzed using a precision‐targeted metabolomics method based on the UPLC‐TQ/MS system (Agilent 1290 Infinity UHPLC and Agilent 6495 QQQ, Agilent Technologies, U.S.A.). Briefly, an ACQUITY UPLC HSS T3 column (2.1 × 100 mm, 1.8 µm) (Waters, USA) was utilized to separate metabolites, and the DMRM scan mode was employed for targeted metabolomics analysis. For more detailed information, please refer to our previous publications. The raw data was centrally processed using the MetaboAnalyst platform [23, 24]

### 2.4 Co‐Analysis of Metagenome and Metabolome

The permutational multivariate analysis of variance (PERMANOVA) test was used to investigate the covariations between differential serum metabolites and the composition of gut microbiota, as well as fecal metabolites [25]. A significance level of *P* < 0.05 was used to indicate a meaningful correlation. Spearman's correlation coefficients were calculated to illustrate the relationships between gut functional pathways and serum metabolites, fecal metabolites, the HH‐N‐enriched species, or the HH‐A‐enriched species. To eliminate false positives, a simple linear regression was conducted on the screened species and metabolites. Associations with *P* > 0.05 were considered as false positives and excluded from further analysis.

### 2.5 Animal Study Design

The fourteen cages were randomly divided into seven groups: the normal atmosphere group (NA), the hyperbaric hypoxic group (HH), the ketoglutaric acid group (HH + α‐KG), the betaine group (HH + Betaine), the L‐aspartic acid group (HH + L‐Asp), the *Streptococcus rubneri* group (HH + *S. rubneri*), and the *Veillonella rogosae* group (HH + *V. rogosae*). Each group consisted of 10 rats (*n* = 10). Following a 7‐day adaptation period to the diet, with the exception of the NA group, the remaining groups were promptly transferred to a hypobaric hypoxia chamber (Shanghai Tawang Intelligent Technology Co., Ltd, China) designed to simulate an altitude of 6000 m for a duration of 60 days.

During the hypobaric hypoxia exposure, the oxygen concentration inside the chamber was maintained at 20% for 24 h, with an oxygen partial pressure of 9.5 kPa. The chamber pressure was set at −58 kPa, while the temperature was maintained at 24°C and the humidity at 60%. The simulated altitude of 6000 m was achieved within 30 min, using a ventilation frequency of 15 times per hour, a duration time of 2 min, an air intake rate of 5 L/min, and an air extraction rate of 20 L/min [13].

In the subsequent 60 days, the chamber was periodically lowered to sea level within a 30‐min timeframe every 3 days at 9:00 a.m. for safety reasons. After the experimental operations, the chamber was re‐elevated to the altitude of 6000 m. The HH + α‐KG group received water supplemented with 1% α‐ketoglutaric acid disodium salt (Shanghai Yuanye Bio‐Technology Co., Ltd), the betaine group received water supplemented with 1% betaine (Shanghai Yuanye Bio‐Technology Co., Ltd), and the HH + L‐Asp group received water supplemented with 1% L‐aspartic acid Mg salt (Shanghai Yuanye Bio‐Technology Co., Ltd). The HH + *V. rogosae* and HH + *S. rubneri* groups were gavaged with 1 × 10^5^ *V. rogosae* and *S. rubneri* every 3 days [26]. The Animal Care and Use Guidelines received approval from the Beijing Institute of Radiation Medicine, with the approval number IACUC DWZX‐2022‐610.

### 2.6 Cell Culture

To induce hypoxia, the cells were exposed to a hypoxia incubator chamber containing a gas mixture of 1% O_2_, 5% CO_2_, and 94% N_2_ for a duration of 24 h. Cell passages 8–10 were utilized for all experimental procedures, ensuring consistency and reliability in the results obtained [27].

### 2.7 Glycolysis Analysis

To examine the influence of different drugs on the glycolytic capacity of cells, a Seahorse XF Glycolytic Rate Assay Kit (Seahorse Bioscience, Agilent) was utilized following the manufacturer's protocols. The Seahorse XF96 Extracellular Flux Analyzer was used to measure the extracellular acidification rate (ECAR). The ECAR value was calculated after normalizing to the cell number and was presented as the mean ± standard deviation (SD) [28].

### 2.8 Statistics

R version 3.4.0, SPSS version 23.0, and GraphPad Prism version 9.5.0 (GraphPad Software) were used for statistical analysis. Data were shown as numbers with percentages or as medians with interquartile ranges. The subgroups and validated cohort distributions were determined by propensity score matching (PSM). Wilcoxon rank‐sum test (two‐tailed) was used to evaluate statistical significance between two groups, with a significance level of *P* < 0.05 considered statistically significant.

Detailed materials and methods are provided in the supplementary information for further details.

## 3 Result

### 3.1 Study Design, Heart Health Monitoring, and Multi‐Omics Profiling

**FIGURE 1:**
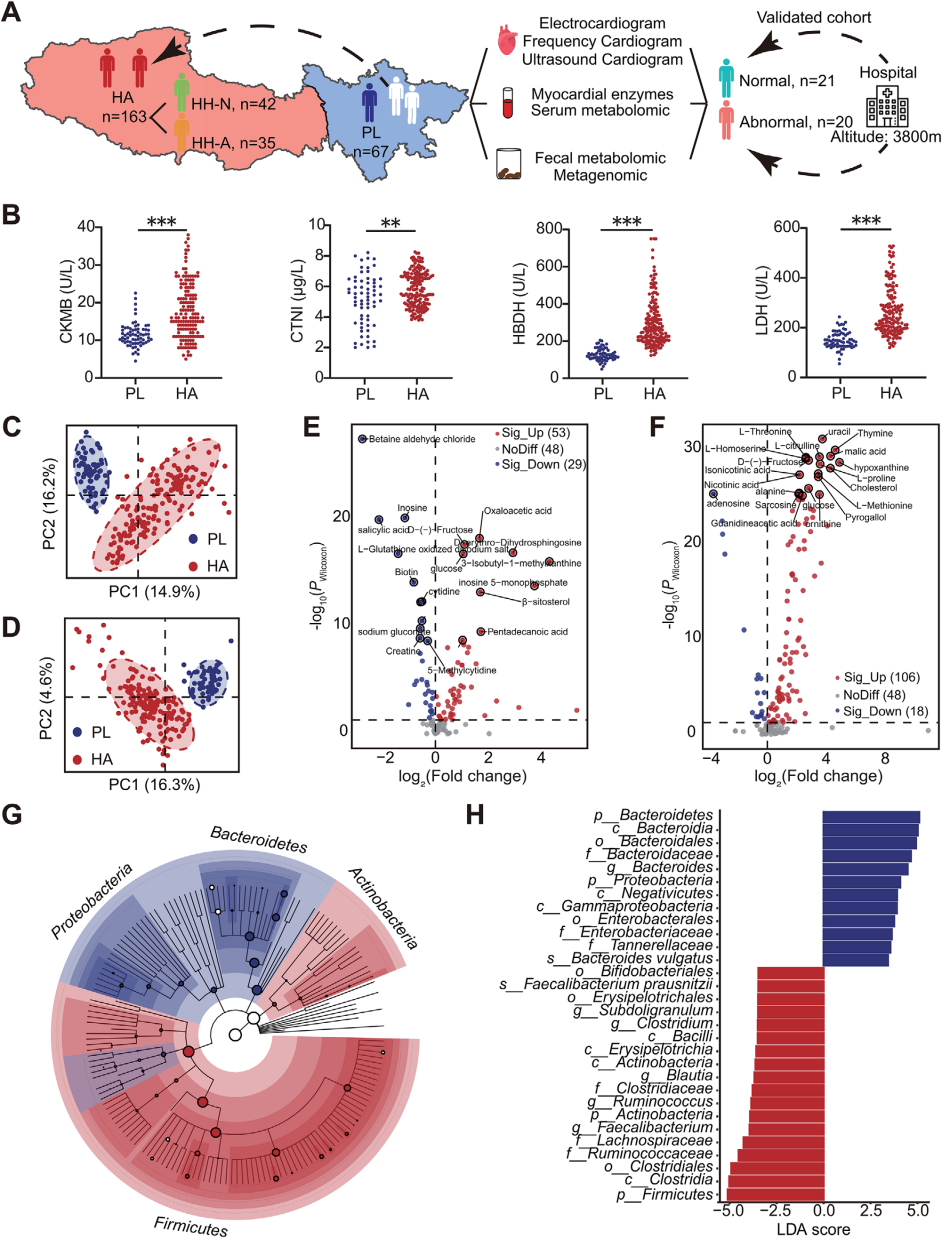
Myocardial enzymes, gut microbial structure, and metabolic concentrations differed between plain controls and plateau migrants. (A) An overview of the workflow for revealing metabolic and microbial features associated with high‐altitude heart abnormalities. (B) Differences in the expression level of myocardial enzymes (CKMB, CTNI, LDH, and HBDH) between PL and HA groups. (C,D) Classification between PL and HA groups revealed by principal component analysis (PCA) based on serum metabolome (C) and fecal metabolome (D). The significance of the results of PCA was assessed by PERMANOVA test. Adonis *p* value = 0.00288 (C), Adonis *p* value = 0.00163 (D). (E,F) Volcano plots for serum metabolites (E) and fecal metabolites (F) between PL and HA groups. The metabolites displaying significant changes (*P* < 0.05) were colored in blue (log_2_(fold change) < 0) and red (log_2_(fold change) > 0). (G) Cladogram of gut microbiota. Each circle represents a taxonomic hierarchy. Dots enriched in PL and HA groups (*P* < 0.05 and |LDA score| > 2) were colored in blue and red, respectively. (H) The bar plot shows the specific gut microbiota with significant differences. The bars colored in blue and red represent gut microbiota enriched in PL and HA groups, respectively. Wilcoxon rank‐sum test was used to detected the differences between groups. *P < 0.05; **P < 0.01; ***P < 0.001. PL group (*n* = 67) and HA group (*n* = 163).

This study included 230 adult males who had graduated from Chengdu Medical College. Among them, 163 individuals had relocated to the Tibetan Plateau for 5 (3, 7) years (referred to as the high‐altitude group, HA), while the remaining 67 individuals continued to reside in Chengdu (referred to as the plain group, PL) (Figure 1A). The two groups were matched in terms of age, BMI, and demographics (Table S1). To detect the effects of high‐altitude environments on heart health, a series of detailed examinations, including ECG, UCG, FCG, and laboratory tests, were conducted for all participants. The results revealed that individuals in the HA group exhibited more frequent abnormal ECG signals characterized by right axis deviation and incomplete right bundle branch block compared to the PL group. Additionally, 12.88% (21/163) and 4.29% (7/163) of plateau migrants showed signs of insufficient perfusion to the myocardium and left ventricle hypertrophy, respectively (Table S1). Elevated levels of myocardial enzymes, such as creatine kinase isoenzymes (CKMB), hydroxybutyrate dehydrogenase (HBDH), lactate dehydrogenase (LDH), and cardiac troponin I (CTNI) (Figure 1B), further indicated a deteriorated heart condition and an increased potential risk of HAHD following prolonged exposure to the high‐altitude hypobaric hypoxic environment.

To better understand the metabolic and microbial characteristics of the two groups, we analyzed their serum and feces metabolome through targeted metabolomics analysis and examined the gut microbiome using metagenomic sequencing of fecal samples. Our data revealed distinct clustering patterns between serum/feces samples from the HA and PL groups (Figure 1C, 1D). A total of 82 and 124 differential metabolites were identified in serum and feces, respectively (Figure S1A,B). Notably, the serum and fecal metabolites in the HA group exhibited a clear increasing trend, with 65% (53/82) of serum metabolites and 85% (106/124) of fecal metabolites showing higher levels compared to the PL group (Figure 1E,F). These differential metabolites were associated with specific metabolic pathways, including aminoacyl tRNA biosynthesis, amino acid metabolism, and the tricarboxylic acid cycle (TCA) (Figure S2A,B).

Moreover, reference‐based metagenomic sequencing revealed significant differences in bacterial alpha and beta diversity between the HA and PL groups (Figure S3A,B), indicating a reduction in bacterial richness but a more uniform community structure among plateau migrants (Figure S3C). Additionally, linear discriminant analysis effect size (Lefse) identified 3172 differential species, with 369 species (Table S4) significantly contributing to the differences in gut microbiota composition between the two groups (*p* < 0.05 and LDA score > 2) (Figure 1G,H). These metagenomic biomarkers predominantly belonged to *Firmicutes*, *Bacteroidetes*, *Actinobacteria*, and *Proteobacteria*, with notable differences in phyla abundances between the HA and PL groups (Figure S3D).

### 3.2 Inter‐Individual Microbiome Structure Variations and the Susceptibility to Undermined Heart Health

**FIGURE 2:**
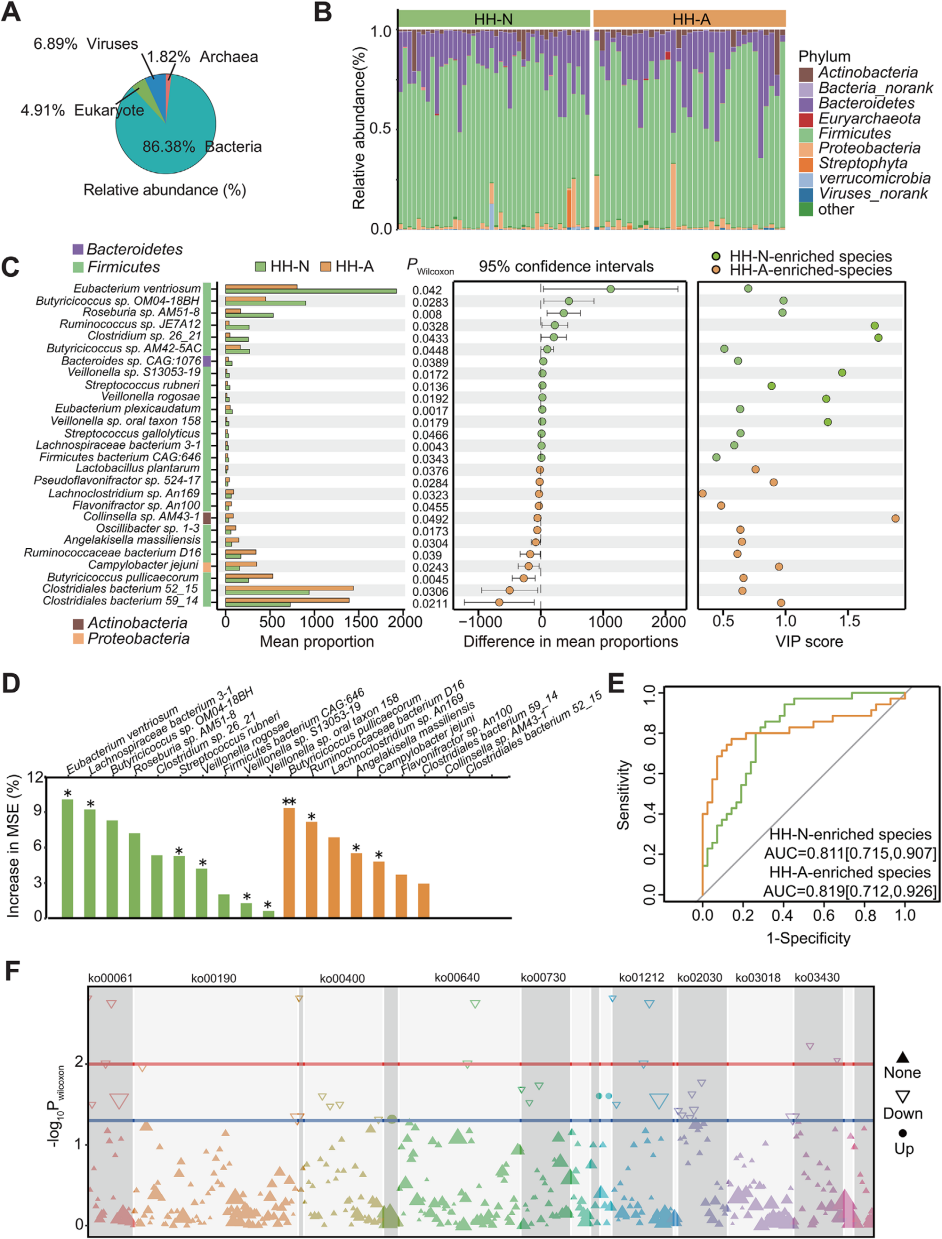
Association of the gut microbial composition and performance with the susceptibility to high‐altitude heart abnormalities. (A) The lineage of gut microbiota based on metagenomics sequencing in the domain level. (B) The composition of gut microbiota at the phylum level in HH‐N and HH‐A groups. (C) Screening of species with differential abundance related to the susceptibility of high‐altitude heart abnormalities. The species with *P* < 0.05 accessed by the Wilcoxon rank‐sum test and VIP score > 1 accessed by partial least squares discriminant analysis (PLS‐DA) was screened. The green and yellow nodes were the HH‐N‐enriched and HH‐A‐enriched species, respectively. (D) The importance of screened species predicted by random forests (RF) in HH‐N and HH‐A groups. The higher mean square error (MSE) value means that the species is more important and can serve as drivers for the prediction of heart health impairment in plateau migrants. Significance was assessed by 1000 randomized permutations and was recorded as follows: *P < 0.05; **P < 0.01; ***P < 0.001. Green, HH‐N‐enriched species; yellow, HH‐A‐enriched species. (E) Classification performance of HH‐N and HH‐A groups by species enriched in each group. The green and yellow lines represent the HH‐N‐enriched and HH‐A‐enriched species, respectively. The AUC value was used to evaluate the performance of each classification. (F) Enriched KEGG pathways according to the gut microbiota with varying abundance between HH‐N and HH‐A groups. Blue line, *P* < 0.05; red line, *P* < 0.01. HH‐N group (*n* = 42), HH‐A group (*n* = 35).

The data mentioned above indicated that not all individuals in the HA group experienced a decline in heart health induced by the hypobaric hypoxia environment, suggesting individual variations in susceptibility to compromised heart health. Increasing evidence has established a significant association between the gut microbiome and the onset and progression of cardiovascular diseases, highlighting the potential role of alterations in gut microbiome composition in heart health among high‐altitude migrants [29]. To pinpoint specific gut microbiome features potentially implicated in the development of compromised heart health in high‐altitude migrants, we categorized individuals in the HA group into two subgroups, HH‐N and HH‐A, based on ECG, FCG, and UCG results. Additionally, given the strong correlations of CKMB values with FCG (*p* = 0.008) and UCG (*p* = 0.002) (Figure S4A), individuals with CKMB values exceeding the clinical reference range (25 U/L) were included in the HH‐A subgroup. Consequently, we enrolled 35 individuals in HH‐A and 42 in HH‐N from the HA group, matched for potential confounders such as age, BMI, smoking history, location altitude, dietary habits, and residence time in Tibet (Table S2). Subsequently, we investigated the gut microbial compositions of the 77 individuals, and totally identified 19902 species that consist of archaea (1.82%), bacteria (86.38%), eukaryotes (4.91%), and viruses (6.89%) (Figure 2A). Discriminative differences in the structure of gut microbiota were observed between the two subgroups (Figure 2B). Compared to HH‐N, individuals in HH‐A exhibited significantly reduced bacterial richness and homogeneity, as confirmed by the Simpson index and Bray–Curtis distance, respectively (Figure S4B,C). After conducting statistical analysis of metagenomic profiles (STAMP) with a significance level of *p* < 0.05, we identified 258 differential species from 19 Phyla (Figure S5–S7, Table S5). Among these, 27 species contributed significantly to the subgroup separation (*P* < 0.05 and VIP score > 1), with 12 species enriched in HH‐A and 15 species enriched in HH‐N (Figure 2C). To further evaluate the importance of the 27 differential species in subgroup separation, we then conducted a tenfold cross‐validation on a random forest model and revealed the top ten species in the two subgroups (Figure 2D). Of these, the abundances of *Eubacterium plexicaudatum, Lachnospiraceae bacterium 3‐1*, *S. rubneri*, *V. rogosae*, *Veillonella sp. oral taxon 158*, and *Veillonella sp. S13053‐19* were lower in HH‐A, but the levels of the other four species (*Angelakisella massiliensis, Butyricicoccus pullicaecorum, Campylobacter jejuni, Ruminococcaceae bacterium D16*) were increased in this subgroup (Figure S4E). Meanwhile, the receiver operating characteristic curve (ROC) analysis showed good efficiency of these ten species as metagenomic biomarkers in the assessment of undermined heart health for plateau migrants, with AUC value of 0.819 (*p* < 0.001, 95%CI (0.712–0.926)) and 0.811 (*p* < 0.001, 95%CI (0.715–0.907)) for species and decreased species, respectively (Figure 2E). Interestingly, the KEGG annotation of metagenome sequences further demonstrated significant enrichment in 24 functional pathways, 13 of which belonged to metabolism‐related category, such as oxidative phosphorylation, amino acid biosynthesis, and fatty acid metabolism (Figure 2F). Therefore, the microbial feature variation might be one of the crucial factors determining the risk to impaired heart health among the high‐altitude migrants, and microbial‐derived or ‐related metabolites could be key elements involved in the pathogenic mechanisms of these adverse outcomes.

### 3.3 Distinct Serum and Fecal Metabolome Signatures Related to Individuals With Undermined Heart Health

**FIGURE 3:**
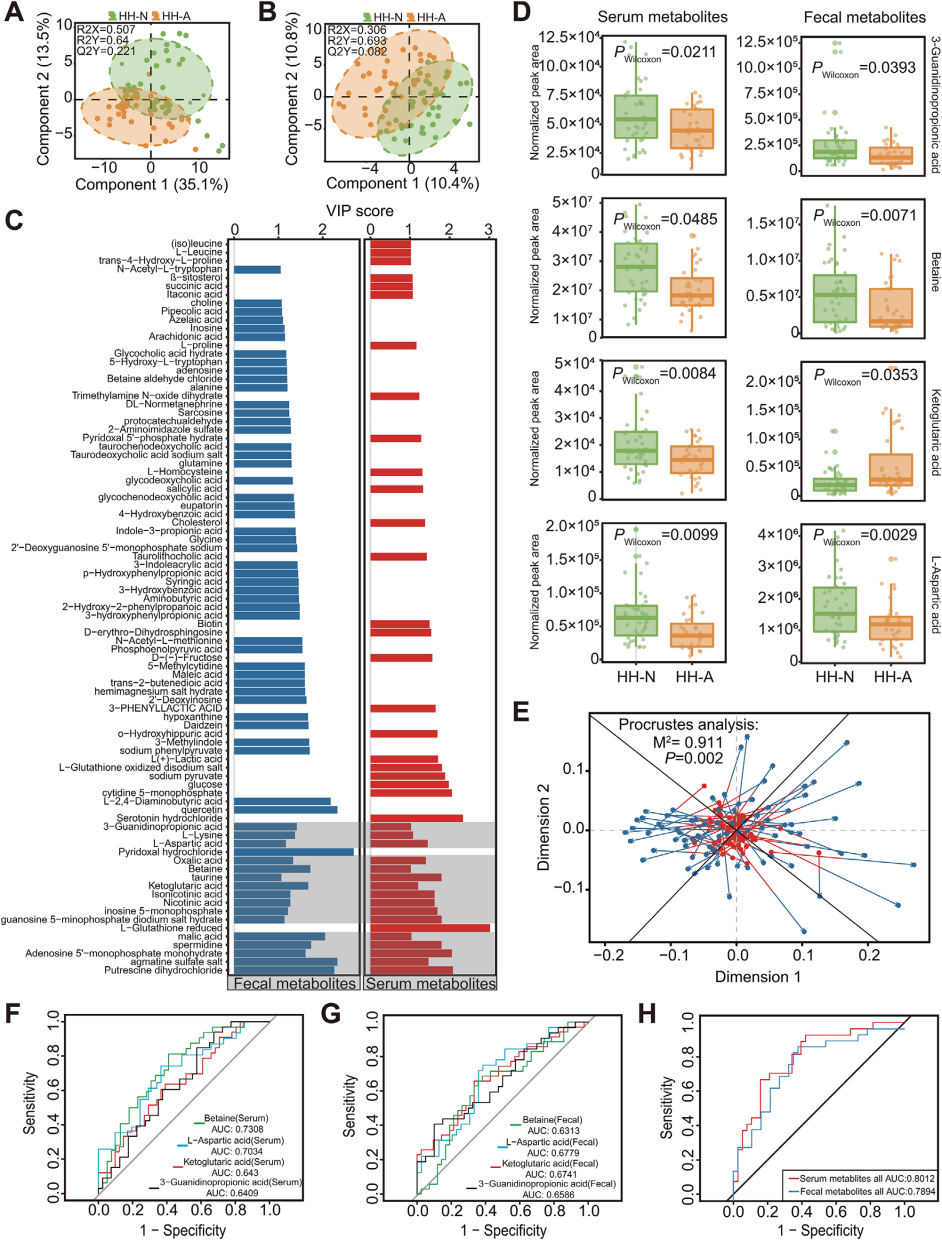
The characteristics of serum and fecal metabolome were related to the individuals with heart abnormalities. (A,B) Classification between the HH‐N and HH‐A groups revealed by PLS‐DA based on serum metabolome (A) and fecal metabolome (B). The *XY*‐axis explanatory degrees and the predictive ability of each model were shown in each subpanel. The green and yellow nodes represent the HH‐N (*n* = 42) and HH‐A (*n* = 35) group, respectively. (C) VIP scores of metabolites in serum and feces. All metabolites were displayed with VIP score > 1. Red bars, serum metabolites; blue bars, fecal metabolites; gray blocks, both serum and fecal metabolites. (D) Abundance differences in metabolites between the HH‐N and HH‐A groups. Wilcoxon rank‐sum test was used to detect the significance of the difference in each comparison. *P < 0.05; **P < 0.01; ***P < 0.001. (E) Procrustes analysis of serum metabolites versus fecal metabolites. Serum and fecal metabolites were shown as red circles and blue circles, respectively. Serum and fecal samples from the same individual were connected by lines. (F,G) Prediction of HAHD by serum metabolites (F) and fecal metabolites (G). The AUC reflects the predictive ability of metabolites with differential abundance for heart abnormalities. The higher the AUC value, the better the predictive ability of the metabolites. (H) Prediction of heart abnormalities by the cluster of serum or fecal metabolites. Serum and fecal metabolites were fitted separately by logistic regression. HH‐N group (*n* = 42), HH‐A group (*n* = 35).

In recent years, scholars have constructed a platform for predicting the association between gut microbial metabolites and diseases, effectively linking gut microbiota to diseases [30]. This study also considers the interplay between host‐microbe co‐metabolism in high‐altitude environments. Consequently, we conducted a comprehensive analysis of serum and fecal metabolomic changes in the cohort of 77 high‐altitude migrants [31]. As anticipated, the serum and fecal metabolite profiles of individuals in the HH‐A subgroup were distinctly separated from those in the HH‐N subgroup (Figure 3A,B). By integrating the VIP plot, we identified that 31% (41 out of 131) of serum metabolites and 36% (62 out of 172) of fecal metabolites significantly contributed to the differentiation between HH‐N and HH‐A subgroups (VIP score > 1, Figure 3C). Among these metabolites, there were 16 overlapping compounds present in both serum and feces (Figure 3C). However, the Wilcoxon rank‐sum test revealed that only four of these overlapping metabolites exhibited significant differential abundances between HH‐N and HH‐A, namely ketoglutaric acid (α‐KG), L‐aspartic (L‐Asp), 3‐guanidinopropionic acid (3‐GUA), betaine (*p* < 0.05, Figure 3D). As for serum metabolome, the abundances of these four metabolites were markedly decreased in the HH‐A subgroup. As for fecal metabolome, the levels of L‐Asp, betaine, and 3‐GUA were also decreased in HH‐A, but α‐KG was enriched in the feces from HH‐A individuals. The results of the procrustes analysis demonstrated a strong correlation among the four metabolites in serum and fecal samples from the 77 individuals (Figure 3E), suggesting a potential relationship between the fecal and serum metabolomes of high‐altitude migrants. Then we conducted ROC analysis to evaluate the accuracy of subgroup discrimination using the four differential metabolites. As shown in Figure 3F–H, the four metabolites (in both serum and feces) together distinguished HH‐A from HH‐N, clearly outperforming L‐Asp, betaine, 3‐GUA, or α‐KG alone. In particular, these four differential metabolites from serum had a higher discrimination accuracy than that in feces, with an AUC of 0.8012. Taken together, our findings the four metabolites could be metabolomic biomarkers related to undermined heart health after high‐altitude settlement.

### 3.4 Gut Microbiome Biomarkers and Associated Serum Metabolites in Detection of Plateau Migrants With Abnormal Heart Health

**FIGURE 4:**
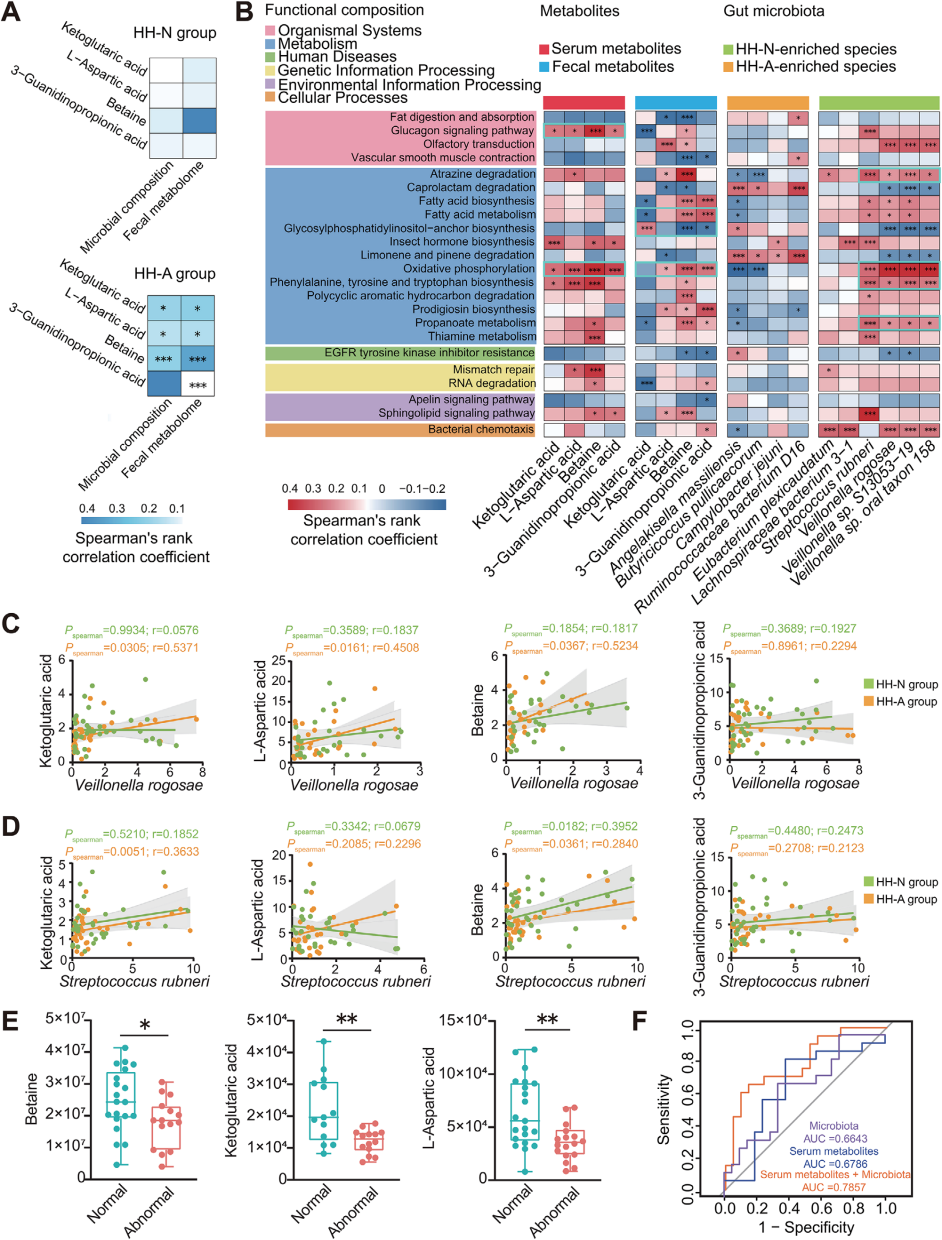
The gut microbiota influences host serum and fecal metabolites in plateau migrants with heart abnormalities. (A) The covariant relationship between each serum metabolite and gut microbial composition or fecal metabolites in HH‐N and HH‐A groups was assessed by PERMANOVA analysis. The effect sizes between serum metabolites and the gut microbiota or fecal metabolites are represented in shades of blue. *P < 0.05; **P < 0.01; ***P < 0.001. (B) The correlation between the concentration of serum and fecal metabolites, HH‐N‐enriched and HH‐A‐enriched species and microbial functions. The heatmap shows the positive correlation with red bars and negative correlation with blue bars. Spearman's rank correlation coefficient was used to show the degree of each correlation. (C,D) Scatterplots showed the correlation between the concentration of each serum metabolite and the abundance of *V. rogosae* (C) or *S. rubneri* (D) in HH‐N and HH‐A groups, respectively. The relationship between the two samples was calculated by Spearman's rank correlation coefficient. (E) Differences in the abundance of L‐Asp, betaine, and α‐KG in validated cohort. Wilcoxon rank‐sum test was used to detect the significance of the difference between each comparison. (F) The ROC curves for demonstrating the predictive ability of selected serum metabolites, gut microbiota and the combination of the two features for heart abnormalities. Blue curve, serum metabolites; purple curve, gut microbiota; orange curve, the combination of the two features. HH‐N group (*n* = 42), HH‐A group (*n* = 35); normal group (*n* = 21), abnormal group (*n* = 20).

To explore the relationships between serum differential metabolites and gut microbial composition or fecal metabolome, we conducted a PERMANOVA test, revealing that the serum differential metabolites were influenced by both microbial composition and fecal metabolome in the HH‐A subgroup (Figure 4A). Subsequently, we examined the correlations between the abundances of the top ten marked microbial species, the concentrations of the four differential metabolites (in both serum and feces), and microbial functions using Spearman correlation analysis. As expected, the enriched species and differential metabolites both had the strongest linkages with metabolic pathway (Figure 4B).

More specifically, the species enriched in HH‐N, including *S. rubneri*, *V. rogosae*, *Veillonella sp. oral taxon 158*, and *Veillonella sp. S13053‐19*, along with the differential metabolites from serum and feces (except for α‐KG in feces), exhibited positive correlations with oxidative phosphorylation. In contrast, significant negative correlations were presented between HH‐A enriched species (*Angelakisella massiliensis* and *Campylobacter jejuni*) and oxidative phosphorylation. Subsequently, the linear regression was performed to detect the correlations between each of the differential metabolites and the two species (*S. rubneri* and *V. rogosae*). As shown in Figure 4C, the abundances of *V. rogosae* were significantly associated with the levels of L‐Asp, betaine, and α‐KG in the serum of HH‐A individuals. And positive correlations between the abundance of *S. rubneri* and the serum levels of betaine and α‐KG were also observed in HH‐A (Figure 4D). But the associations of the two species and the serum concentration of 3‐GUA were not significant. Hence, the alterations in microbiome biomarkers and serum metabolites, including L‐Asp, betaine, and α‐KG which together are termed as the GMSM panel, could be multi‐omics signatures of HH‐A individuals.

To further validate our gut microbial and metabolic findings, we assembled a cohort of 41 high‐altitude migrants, comprising 20 individuals with abnormal heart health and 21 normal participants, carefully matched for smoking history, location altitude, dietary habits, and residence time in Tibet (Table S3). This independent validation cohort underwent analysis of the serum and fecal metabolome, as well as the gut microbiome, utilizing targeted metabolite profiling and metagenomic sequencing. In line with the results from the initial cohort, individuals with abnormal heart health exhibited significant reductions in serum levels of L‐Asp, betaine, and α‐KG (Figure 4E). Similarly, evident decreases in the abundances of *S. rubneri* and *V. rogosae* were observed in the abnormal subpopulation (Figure S8). Similarly, there were noticeable decreases in the abundances of *S. rubneri* and *V. rogosae* within the subgroup with abnormal heart health (Figure S8). While the majority of species (6 out of 10) showed statistical significance (*p* < 0.05) (Figure S8), not all species reached this threshold, likely due to limitations in cohort size. To further assess the diagnostic efficacy of these microbiome biomarkers and the GMSM panel, we employed random forest classifiers to distinguish high‐altitude migrants with abnormal heart health from matched healthy controls. The ROC curve illustrated that the combination of microbiome biomarkers and the GMSM panel outperformed the use of microbiome biomarkers or the GMSM panel alone in identifying heart health abnormalities in plateau migrants, with an elevated AUC value of 0.7857 (Figure 4F). Collectively, our results indicated that the altered microbial and metabolic biomarkers could be diagnostic markers or classifiers to recognize HH‐A individuals.

### 3.5 Supplementation With *S. rubneri*, *V. rogosae*, or Associated Serum Metabolites Attenuated Hypobaric Hypoxia‐Induced Cardiac Hypertrophy in Rodents

**FIGURE 5:**
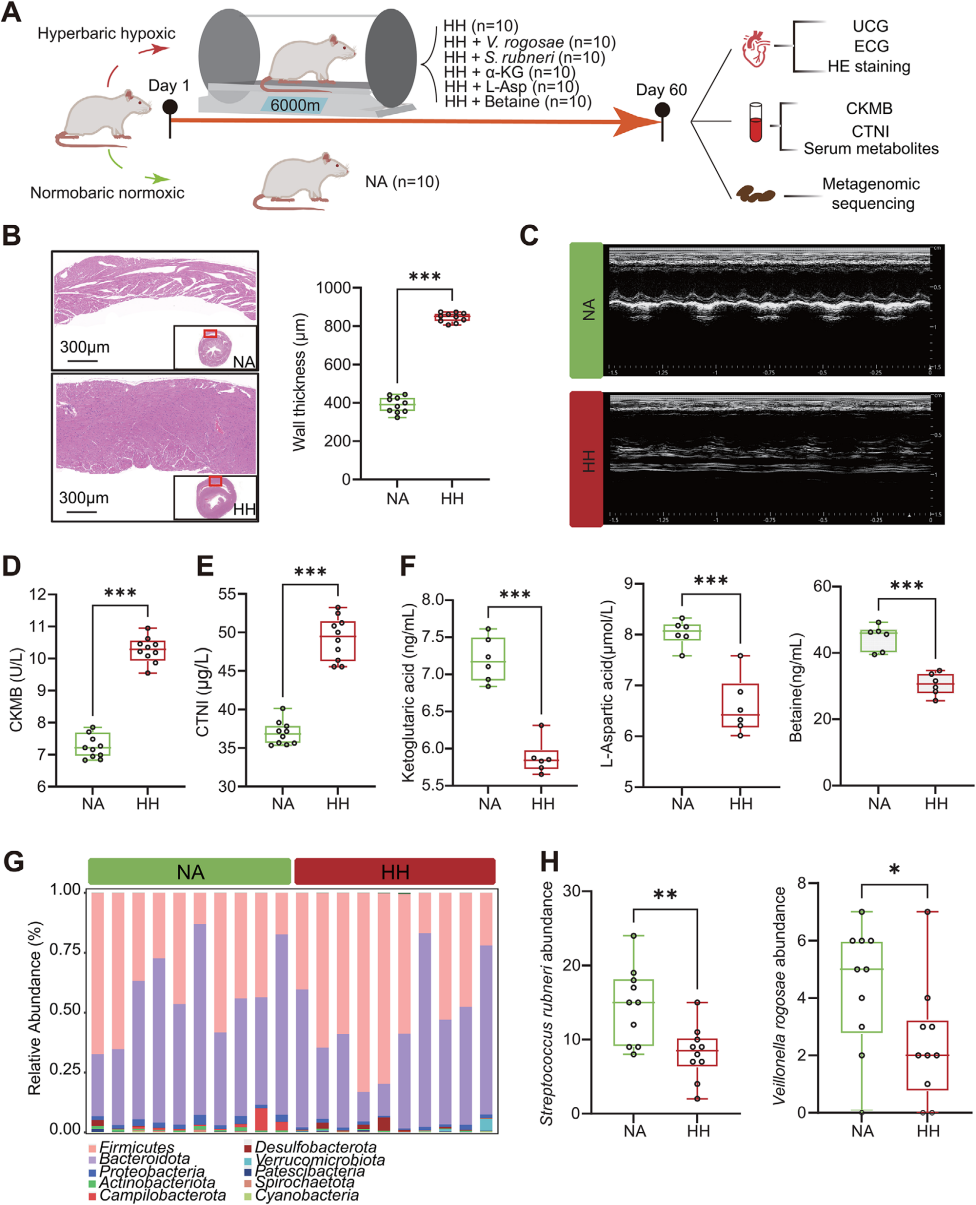
Hypobaric hypoxia affects heart structure, myocardial‐enzymes values, gut microbial composition, and metabolic concentrations in rats. (A) Flow chart of rat experiments. Seventy rats were equally divided into seven groups: Group 1, the NA group; Groups 2–7, the hypobaric hypoxic chamber groups, of which one group was the HH group and the remaining five groups were the intervention groups. (B) Hematoxylin‐Eosin (HE) staining demonstrated that the wall thickness in rat hearts changed under hypobaric hypoxic environment. (C) The examinations of UCG in rats under hypobaric hypoxic environment. (D,E) Differences in the expression level of CKMB (D) and CTNI (E) between NA (*n* = 10) and HH (*n* = 10) groups. (F) Differences in the abundance of the serum metabolites (L‐Asp, betaine and α‐KG) between NA (*n* = 6) and HH (*n* = 6) groups. (G) The composition of gut microbiota at phylum level in NA (*n* = 10) and HH (*n* = 10) groups. (H) Differences in the abundance of *V. rogosae* and *S. rubneri* between NA (*n* = 10) and HH (*n* = 10) groups. *P < 0.05; **P < 0.01; ***P < 0.001. The green box represents the NA group and the red box represents the HH group.

To identify the specific biological effects of the altered microbial species and GMSMs, we next carried out in vivo experiments according to the schedule presented in Figure 5A. After a 60‐day exposure to a hypobaric hypoxia chamber simulating an altitude of 6000 m with 9.5 kPa atmospheric pressure, the rats indeed suffered from remarkable pathological cardiac hypertrophy (Figure 5B), both of which were confirmed by elevated ECG and UCG examinations (Figure 5C and Figure S9A–C) as well as increased serum levels of CTNI and CKMB (Figure 5D,E). Moreover, the tendencies of hypobaric hypoxia‐induced alterations in the concentrations of GMSMs in rat serum coincided exactly with those in the abovementioned cohort studies (Figure 5F). Subsequent metagenomic sequencing of rat stool samples from the HH and NA groups revealed a notable shift in gut microbial composition at the phylum level due to hypobaric hypoxia exposure, consistent with the findings in the cohort study. This shift was characterized by an increase in *Firmicutes* but decreases in *Actinobacteria*, *Bacteroidota*, and *Proteobacteria* (Figure 5G and Figure S9D). Also, long‐term living in a hypobaric hypoxia chamber significantly decreased the abundances of *V. rogosae* and *S. rubneri* at the species level (Figure 5H).

**FIGURE 6:**
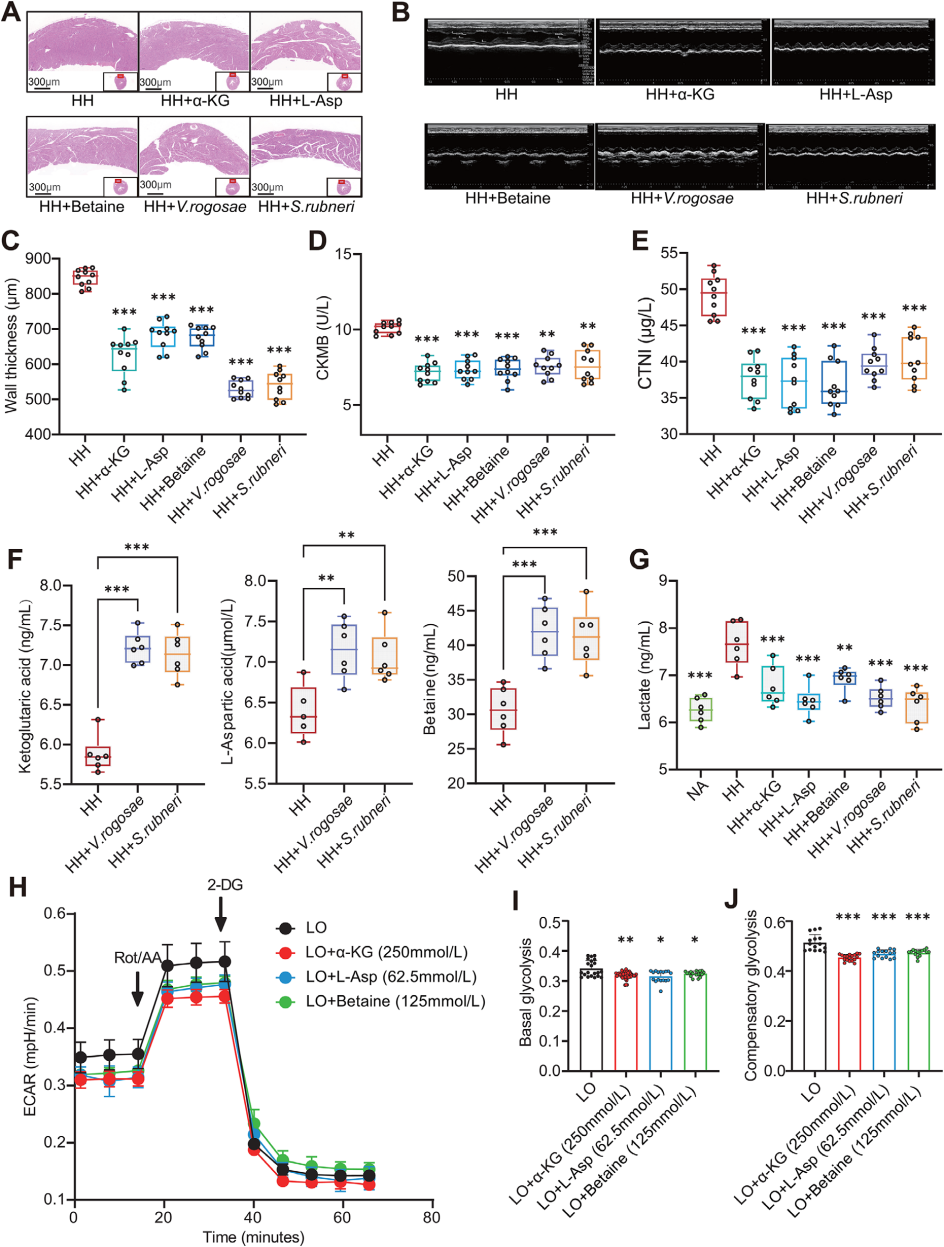
*V. rogosae*, *S. rubneri*, and metabolites (L‐Asp, betaine, and α‐KG) attenuated heart hypertrophy in rats. (A) HE staining showed changes in wall thickness in rat hearts fed with or without *V. rogosae*, *S. rubneri*, and metabolites (L‐Asp, betaine, and α‐KG) under hypobaric hypoxic environment. (B) The examinations of UCG in rat hearts fed with or without *V. rogosae*, *S. rubneri*, and serum metabolites (L‐Asp, betaine, and α‐KG) under hypobaric hypoxic environment. (C–E) The differences in the wall thickness of heart (C), the expression level of CKMB (D), and CTNI values (E) among HH and intervention groups (The sample size per group is 10.). (F) Changes in the concentration of serum metabolites (L‐Asp, betaine, and α‐KG) fed with *V. rogosae* and *S. rubneri* (The sample size per group is 6.). (G) Differences in the concentration of lactate in serum among NA, HH and intervention groups (The sample size per group is 6.). (H–J) The effect of L‐Asp, betaine, and α‐KG on the glycolysis rate (H), including basal glycolysis rate (I) and compensatory glycolysis rate (J) tested by ECAR in AC16 cells. *P < 0.05; **P < 0.01; ***P < 0.001.

Meanwhile, we added each of the GMSMs into drinking water or intestinally supplemented with the two species every 3 days, and found elevated contents of myocardial enzymes, morphologic and electrocardiosignal abnormalities, and thickened left ventricular walls caused by hypobaric hypoxia were both significantly alleviated by the two intestinal bacteria and GMSMs (Figure 6A–E and Figure S10). Besides, treatment with the two gut bacteria could rectify hypobaric hypoxia‐induced disorders of GMSM in rat serum (Figure 6F), directly indicating that the production of these metabolites could be gut‐microbiota‐associated. Furthermore, the serum levels of lactate in HH‐A individuals were found to substantially more than that in HH‐N individuals (Figure S4D), and in vivo experiments conducted on rats in the hypobaric hypoxia chamber also revealed an increase in serum lactate levels in the HH group (Figure 6G), probably because enhanced glycolysis under low‐oxygen condition. Interestingly, *S. rubneri, V. rogosae*, and the three metabolites both markedly suppressed the occurrence of higher levels of serum lactate in rodent models after 60 days’ exposure to hypobaric hypoxia (Figure 6G). Moreover, the glycolytic capacity of myocardial cells exposed to low oxygen, including basal glycolysis and compensatory glycolysis, was significantly inhibited by the two bacteria and the GMSM (Figure 6H–J). Consequently, these results indicated that the gut microbiome and GMSM could improve cardiac health, possibly via suppressing hypoxia‐triggered glycolysis and the accumulation of lactate.

### 3.6 Integrated Analysis of the Microbial Mechanisms Underlying Metabolic Processes Regulation in HH‐A Individuals

Multiple lines of evidence have manifested that various metabolic processes were most likely affected by structural alterations in intestinal microbial composition in HH‐A individuals. Metagenomic and metabolomic analysis detected 199 species, seven enzymes, and eight circulating metabolites in HH‐A individuals corresponding to the outlined KEGG pathways, including glycolysis, TCA, and oxidative phosphorylation.

**FIGURE 7:**
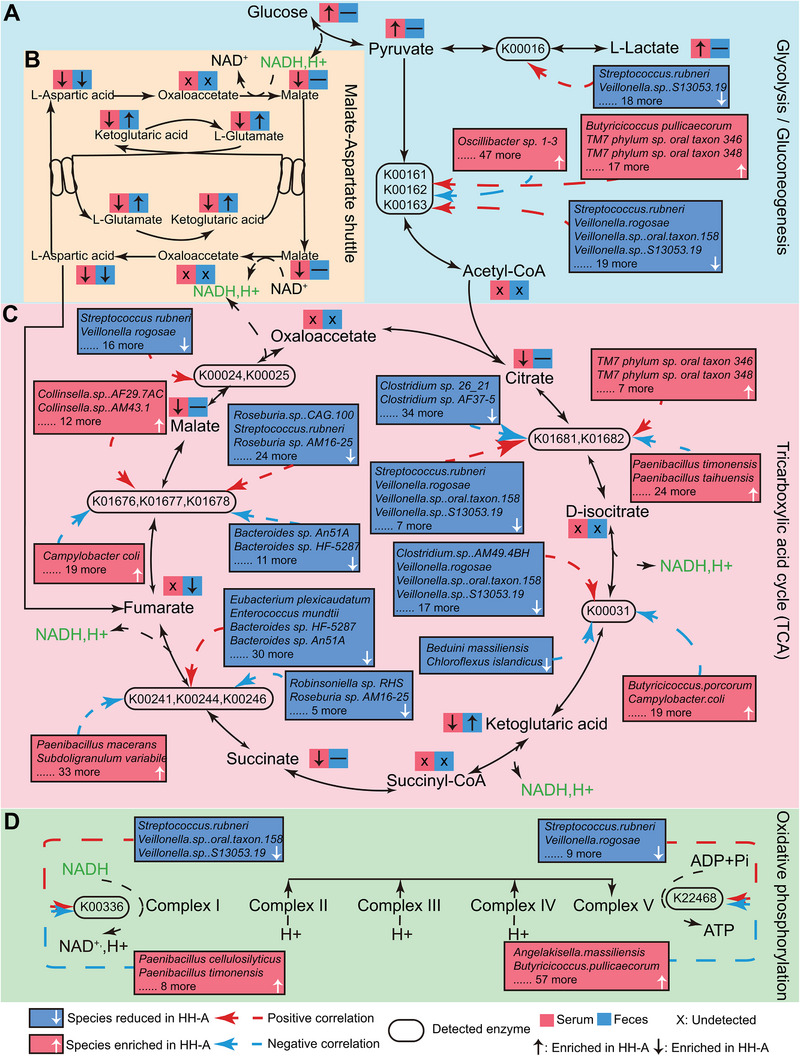
Putative mechanisms linking gut microbiota and metabolites to the susceptibility of heart health abnormalities. (A–D) The inhibition of gut oxidative phosphorylation (D) was positively correlated with the decrease of the abundance of HH‐N‐enriched species, and the NADH required in mitochondria was reduced, resulting in the reduction of the level of MA shuttle (B) and TCA (C), and the concentrations of related serum metabolites (L‐Asp, α‐KG). Due to the decreased concentrations of TCA (C) and MA shuttle (B) substrate (L‐Asp, α‐KG), myocardial cells were mainly powered by glycolysis (A), the gluconeogenesis pathway was inhibited (A), lactate accumulation, and myocardial cells were damaged. Blue boxes with write downward arrow indicate species reduced in HH‐A group. Red boxes with write upward arrow indicate species enriched in HH‐A group. Red boxes with black downward arrows, upward arrows, horizontal lines, and crosses indicate that serum metabolites were decreased, increased, unchanged, and not detected in the HH‐A group, respectively. Blue boxes with black downward arrows, upward arrows, horizontal lines, and crosses indicate that fecal metabolites were decreased, increased, unchanged, and not detected in the HH‐A group, respectively. The red and blue dashed arrows indicate that the species are positively or negatively correlated with the expression level of enzymes, respectively.

As shown in Figure 7A, up to 47 species that increased in HH‐A individuals had negative effects on pyruvate dehydrogenase (K00241, K00244, and K00246). And another 17 elevated species and 19 reduced species (including *S. rubneri*, *V. rogosae*, *Veillonella sp. oral taxon 158*, and *Veillonella sp. S13053‐19*) both showed positive effects on this enzyme. Accompanied by up‐regulated circulating pyruvate and lactate, indicating obvious enhancement in glycolysis in HH‐A individuals. Interestingly, we also found significant declines in circulating L‐Asp, α‐KG, L‐Glutamate, and Malate, all of which were key elements of the malate‐aspartate (MA) shuttle (Figure 7B).

Except for pyruvate dehydrogenase, the other four critical enzymes in TCA, including cis‐aconitase (K01681 and K01682), isocitrate dehydrogenase (K00031), succinodehydrogenase (K00241, K00244, and K00246) and fumarase (K01676, K01677, and K01678), were both speculated to be affected by altered gut bacteria (Figure 7C). Mostly, these species that were positively associated with these enzymes significantly reduced in the HH‐A individuals, like *S. rubneri*, *V. rogosae*, *Veillonella sp. oral taxon 158*, and *Veillonella sp. S13053‐19*. Inversely, negative relationships between the majority of enhanced species and these enzymes were uncovered, such as *Campylobacter coli*. Concomitantly, we also detected prominent reductions in circulating citrate, α‐KG, succinate, and malate, suggesting the suppression of TCA in the HH‐A individuals. As expected, NADH‐quinone oxidoreductase (K00336) and polyphosphokinase (K22468), which are respectively located at respiratory chain complex I and cytochrome oxidase (complex V), were negatively regulated by enriched species but positively associated with the decreased gut bacteria in the HH‐A individuals (Figure 7D). Collectively, our results indicated the critical role of altered gut‐microbiota in metabolic processes remodeling, and suggested that the microbial facilitated glycolysis would participate in high‐altitude‐associated heart health abnormality.

## 4 Discussion

Young individuals migrating to high altitudes exhibit varying susceptibilities to HAHD, often triggered by maladaptation to hypobaric hypoxic environments. Recent studies have suggested that enteric dysbiosis and alterations in related metabolites play causal roles in cardiovascular disease, particularly in its early stages. However, studies focusing on HAHD‐specific gut microbiome and microbial‐associated metabolome signatures are scarce. Our study demonstrated that, compared to the PL group, residing at high altitudes for several years resulted in significant differences in the gut microbial community structure and metabolic features in the serum and feces of HA individuals. Notably, multi‐omics integration analysis first revealed that significant decreases in two taxonomic biomarkers (*S. rubneri* and *V. rogosae*) and three gut microbiota‐associated metabolites (L‐Asp, betaine, and α‐KG, collectively termed the GMSM panel) were strongly associated with heart health abnormalities in plateau migrants. Supplementation with the two species or the GMSMs effectively alleviated hypobaric hypoxia‐induced cardiac hypertrophy and myocardial injury in rodent models. Mechanistically, the transformation of metabolic processes resulting from reductions in these GMSMs, including elevated glycolysis and subsequent serum lactate accumulation, along with the inhibition of the MA shuttle, TCA cycle, and oxidative phosphorylation, could ultimately promote heart health impairment. Our study suggests that differences in gut microbiota communities and gut microbiota‐associated metabolites likely determine susceptibility to heart health abnormalities and HAHD in young plateau migrants.

Recent studies have shown that exposure to high‐altitude environments can lead to structural disorders of the human gut microbiota, with significant decreases in richness and diversity [32], consistent with our findings. The disorder of gut microbiota may result from changes in intestinal blood supply and damage to the intestinal barrier due to hypoxia, which leads to physiological stress in the intestine [33]. Additionally, the inability to adapt to high‐altitude diets may contribute to the limited reproduction of species that are adapted to high‐altitude environments [34]. Moreover, exposure to high‐altitude environments can influence the structure of gut microbiota during the adaptation process due to the colonization of numerous high‐altitude‐specific microorganisms in the intestine. At the class level, we observed a higher abundance of *Clostridia* in the HA group, which has been shown to cause damage to the cardiovascular system. In the plateau migrant population, elevated abundances of *Erysipelotrichaceae*, *Ruminococcaceae*, and *Lachnospiraceae*, and decreased abundance of *Enterobacterales* were identified, all of which have been reported to play important roles in alleviating heart inflammation [35, 36]. Overall, most changes in microbial composition were associated with cardiovascular health. The heart, known to be vulnerable to hypobaric hypoxia, was found to be affected in some plateau migrants, suggesting potential diversity in microbial community structure shifts [8]. The following subgroup analysis confirmed that the bacterial richness and homogeneity among the individuals in HH‐A were both significantly reduced. Notably, the abundances of *V. rogosae* and *S. rubneri* were first found to decrease in HH‐A group, and exhibited excellent contributions to the subgroup separation. In previous articles, these two genera in which the two species belong were identified in the gastrointestinal tract of infants and exhibited the capacity to metabolize sugar, thereby supplying essential nutrients and energy to the body [37]. Moreover, certain strains of *Veillonella* were observed to be involved in the entire lactate metabolism pathway, facilitating the breakdown of lactate generated under anaerobic conditions [38]. But the two species had not been reported to be cardiovascular diseases linked. Intriguingly, supplement with *V. rogosae* and *S. rubneri* significantly attenuated high‐altitude exposure‐induced heart health impairment in rats, first confirming the cardiac protective role of these gut bacteria for plateau migrants.

Given that metabolites mediate interactions between gut microbiota and the host, this study further analyzed serum and fecal metabolome features to detect the pathogenic mechanisms of high‐altitude‐associated heart health abnormalities under conditions of reduced *V. rogosae* and *S. rubneri* abundance. Unlike commonly discussed microbial‐derived or ‐related metabolites involved in cardiovascular diseases, such as SCFAs and trimethylamine N‐oxide (TMAO) [39], the serum levels of three metabolites (L‐Asp, betaine, and α‐KG) significantly reduced in the HH‐A group were found to be related to compromised heart health after high‐altitude settlement. L‐Asp has been shown to improve myocardial contraction by delivering electrolytes to the myocardium [40]. Betaine supplementation has anti‐inflammatory effects and can effectively improve abnormalities in right ventricular systolic pressure, mean pulmonary arterial pressure, right ventricle hypertrophy index, and pulmonary arterial remodeling induced by monocrotaline [41]. α‐KG can prevent myocardial apoptosis and oxidative damage, and attenuate the decline in cardiac function and myocardial fibrosis [42, 43]. Recent studies have revealed that screening the metabolites existing commonly in multiple beneficial (or negatively associated) bacteria through bioinformatics methods can be employed for the selection of candidate drugs for specific diseases [44]. This study confirmed that these three metabolites could effectively improve heart health in rats under hypobaric hypoxia conditions. Surprisingly, supplementation with *V. rogosae* and *S. rubneri* also enhanced the serum levels of these metabolites. Combined with the positive relationships between the three metabolites and the two bacteria at both content and functional levels, our results first revealed that L‐Asp, betaine, and α‐KG could be associated with these two species. These findings imply that high‐altitude exposure leading to reductions in *V. rogosae* and *S. rubneri* abundances contributes to heart health declines in plateau migrants, possibly due to significant decreases in their GMSM levels. Notably, *V. rogosae, S. rubneri*, and their GMSM panel together act as multi‐omics signatures of HH‐A individuals, validated in an independent cohort of this study.

Generally, the heart has a developed myofibril and aerobic metabolic system, with fatty acids providing up to 70% of the energy for cardiac contraction and circulation [45]. Upon acute exposure to a hypoxic environment, glucose provides a rapid ATP supply for cardiomyocytes via glycolysis [46]. But on the downside, this anaerobic metabolic pattern would bring remarkable lactate production and accumulation, which in turn could induce myocardial damages [47]. Therefore, in the short term, enhanced heart rate and blood circulation provide sufficient oxygen to the heart. When the body adapts to high‐altitude environment, a slower heart rate that even lower than the plain level would ease the cardiac burden under the limited oxygen supply condition, eventually bringing a protective effect on the heart [48]. All of these adaptive responses aim to ensure the normal operation of aerobic metabolism in cardiomyocytes of plateau migrants. However, the multi‐omics analysis in this study revealed that identified gut bacteria and GMSMs were positive related to the functions of TCA and oxidative phosphorylation, both of which are the crucial processes of aerobic metabolism. For one thing, HH‐A individuals had much lower contents of α‐KG, which is a TCA intermediate that controls the oxidative synthesis of succinyl‐CoA and following ATP production, accompanying with significant decreases in other circulating important intermediates in the citric acid cycle like citrate and succinate [49], indicating obvious suppression of TCA process in the plateau migrants with heart health abnormality. Moreover, the reductions of circulating L‐Asp, α‐KG, L‐Glutamate, and Malate, all of which together participate in the MA shuttle, could impede the transfer of NADH into the mitochondria, resulting in the discount of the ATP production derived from glycolysis [50]. In this case, more glycolysis happened, meaning more lactate accumulation, especially in the absence of TCA for lactate clearance.

Recent studies have demonstrated that gut microbiota‐derived enzymes, particularly microbial‐host isozymes, might influence host physiology [51]. As shown in our study, the content of pyruvate dehydrogenase was inhibited in plateau migrants with declining heart health due to significant reductions in the abundances of *V. rogosae, S. rubneri*, and other 17 species. This would directly lower the content of acetyl‐CoA entering TCA. Moreover, the gut microbial community in these individuals was prone to exert negative effects on key enzymes involved in TCA, thereby decreasing the energy supply via aerobic oxidation. In addition, NADH produced during glycolysis or TCA is catalyzed to generate ATP through the respiratory chain located at the mitochondrial inner membrane. NADH dehydrogenase, known as complex I, is one of the four main complexes of the respiratory chain and is responsible for electron transfer from NADH/NADPH to coenzyme Q, which is a critical step in the production of ATP via oxidative phosphorylation [52]. Besides, ATP synthase is also called complex V which finally drives ATP generation through the respiratory chain electron transport process coupled ADP phosphorylation [53]. Unfortunately, correlation analysis showed the intestinal bacterial composition in HH‐A individuals, like lower abundances of *V. rogosae* and *S. rubneri*, obviously down‐regulated the contents of NADH dehydrogenase and ATP synthase, suggesting the remarkable suppressions of oxidative phosphorylation among these part of plateau migrants. Taken together, the decreased abundances of protective gut microbes (such as *V. rogosae* and *S. rubneri*) and their GMSMs negatively regulated biological oxidation, including TCA and oxidative phosphorylation. Consequently, cardiomyocytes turned to elevate the proportion of anaerobic metabolism, leading to cardiac functional or structural abnormality.

In summary, plateau migration brought immense changes in the features of the gut microbiome and the serum and fecal metabolome. The primary factors contributing to this difference include alterations in co‐metabolism patterns and substrates between the host and gut microbiota in high‐altitude environments, as well as disruptions in gut microbiota balance and changes in intestinal wall barrier permeability induced by high‐altitude‐specific microorganisms. Research has shown that high‐altitude environments can reshape the energy metabolism patterns of both the body and gut microbiota, with high‐altitude regions primarily characterized by high‐protein and high‐fat diets, resulting in variations in gut microbiota structure, serum composition, and fecal metabolites among young immigrants [54]. Additionally, exposure to high‐altitude environments results in the colonization of plateau‐specific microorganisms in the gut, facilitating adaptation to environmental and dietary changes [55]. Moreover, alterations in immunity and the turnover rate of intestinal epithelial cells contribute to increased permeability of the intestinal barrier and enhanced exchange of metabolites between capillaries and the intestine [56]. In our study, the reduced abundances of two bacterial species (*V. rogosae* and *S. rubneri*) and the lowered contents of their associated metabolites, including L‐Asp, α‐KG, and betaine, were identified to be positively related to heart health abnormality in young migrants at high‐altitude. To a certain extent, the decreases in the concentrations of these GMSMs significantly affected the MA shuttle, TCA, and oxidative phosphorylation, and forced cellular metabolic processes into a facilitated glycolysis status, resulting lactate accumulation and an acid cellular environment. Eventually, these alterations likely enhanced the myocardial maladaptation to hypobaric hypoxia, and then caused heart health abnormality and even the onset of HAHD. Taken together, *V. rogosae*, *S. rubneri*, and the GMSM panel could function as the microbiome biomarkers and the metabolic biomarkers, respectively, both of which not only could be used to early diagnosis of heart health impairment caused by high‐altitude environment, but also provide potential therapeutic interventions for HAHD.

## Author Contributions

Y.G., W.Z., H.L. and B.H. conceived the study and supervised the project; W.Z., Y.Z., Z.N., P.S., and D.S. wrote the manuscript; W.Z., X.C., P.S., Z.B., Y.H., Y.W., and X.T. completed the cohorts and data collection; R.W. and L.G. performed the clinical examination. Y.Z., P.S., D.S., and Y.L. performed metagenomic sequencing and data analysis; J.L. performed metabolome profiling and data analysis; Y.Z., Z.N., G.L., and N.W. completed the animal experiments. All authors have read and approved the final version of the manuscript.

## Conflicts of Interest

The authors declare no conflicts of interest.

## Supporting information




**Supporting File 1**: exp270063‐sup‐0001‐SuppMat.pdf

## Data Availability

All sequencing profiles, including metagenomic sequencing and metabolome sequencing (serum and feces), were uploaded to NCBI (PRJNA1010859 and PRJNA1010875) and Metabolomics workbench (Data track ID: 4270, 4271), respectively.
