# Supplementary material for: Gut Microbiota‐Associated Metabolites Affected the Susceptibility to Heart Health Abnormality in Young Migrants at High‐Altitude: Gut Microbiota and Associated Metabolites Impart Heart Health in Plateau
Source: Exploration (Beijing). 2025 Jun 12;5(4):20240332. doi: 10.1002/EXP.20240332 (PMC12380068; doi:10.1002/EXP.20240332)
Supplement: Supplementary file 1 — Supporting File 1: exp270063‐sup‐0001‐SuppMat.pdf [file EXP2-5-20240332-s001.pdf]

Detail materials and methods

Cohort description and sample collection

All participants completed a comprehensive basic information questionnaire, which included details such as age, educational attainment, location altitude, residence time in Tibet (for plateau migrants only), body mass index (BMI), history of smoking and alcohol consumption, history of antibiotic usage, and past medical history. The inclusion criteria involved the selection of participants aged between 20 and 30, with no prior history of heart disease or a family history indicating genetic predisposition. The exclusion criteria covered individuals who had taken antibiotics, undergone bariatric surgery or intestinal resection within the past 3 months (excluding appendectomy), had inflammatory bowel disease or autoimmune disease, were affected by infectious diseases (such as hepatitis B or C, or human immunodeficiency viruses), had a history of organ transplantation or were on immunosuppressive therapy, or were dealing with drug or alcohol addiction.

Blood samples were obtained from both groups using evacuated blood collection tubes (Becton Dickinson MEDICAL DEVICES (Shanghai) Co., Ltd). The samples were left undisturbed for 30 minutes and then subjected to centrifugation at 5000 rpm/min for 15 minutes at a temperature of 4°C. From each sample, 0.5ml of serum was extracted for biochemical testing, which included parameters such as CKMB, HBDH, LDH, and CTNI. A Roche c311 biochemical assay (Shenzhen Roche Biotechnology Co., Ltd.) was utilized for conducting these tests. The remaining samples were stored at -80°C for subsequent serum metabolomic profiling. Fecal samples were collected from participants using fecal sampling tubes (Shenzhen Medico Biomedical Technology Co., Ltd) and were also stored at -80°C after sampling. These samples were utilized for metagenomic sequencing and metabolomic profiling.

Metagenomic analysis

Alpha diversity was evaluated by the Shannon and Simpson indices. Bray-Curtis and Euclidean distances were applied to assess the variations in species abundance between two groups. The Analysis of Similarities (ANOSIM) was performed to determine the significance index (P), with a threshold of $P < 0.05$ indicating significant differences in species abundance between the groups. At the domain level, the Wilcoxon rank-sum test was used to demonstrate differences in the abundance of archaea, bacteria, eukaryotes and viruses between the two groups. The top ten phyla were obtained by calculating the relative abundance of species at the phylum level in both groups. Linear discriminant analysis effect size (Lefse) was employed to identify the differences in the abundance of microbial features. Differential species were filtered by Partial Least Squares Discriminant Analysis (PLS-DA) with VIP Score >1 and Stamp analysis with $P < 0.05$ using STAMP 2.1.3. Furthermore, the importance of species in grouping was evaluated through ten-fold cross-validated classification using random forest. The significance of importance scores was obtained through 1,000-times permutation analysis. Based on the abundance

difference between the two groups, species screened were divided into HH-N-enriched and HH-A-enriched groups. ROC analysis was conducted to assess the predictive effect on the disease. To display differential functional pathways and enzymes, a Manhattan plot was employed. Associations between the gut microbiota and enzymes or functional pathways were examined using Spearman's correlation coefficients.

Metabolome analysis

Significant metabolites were identified using pls-da analysis with VIP Score >1. Metabolites meeting the VIP Score threshold in both fecal and serum samples were selected. Differences in metabolite concentrations between the two groups were assessed using the Wilcoxon rank-sum test. To evaluate the association between serum and fecal metabolites, Procrustes analysis was performed. The significant correlations were assessed by PERMANOVA test, with a significance threshold of $P < 0.05$ indicating an association between serum fecal metabolites. The predictive potential of metabolites for disease was evaluated using ROC curves.

Bacterial strains and culture conditions

V.rogosae (Taxonomy ID: 423477) was acquired from the Japan Collection of Microorganisms (Tsukuba, Japan) and cultivated using a 2.5L round bottom vertical anaerobic culture bag (Qingdao Haibo Biotechnology Co., Ltd, China) in conjunction with an anaerobic gas-producing bag (Qingdao Haibo Biotechnology Co., Ltd, China). The culture medium used was medium 14 from the Beijing BeNa Culture Collection.

S.rubneri (Taxonomy ID: 1234680) was obtained from the BeNa Culture Collection (Beijing, China) and maintained using a 2.5L round bottom vertical anaerobic culture bag (Qingdao Haibo Biotechnology Co., Ltd, China) with the assistance of AraeroPack-MicroAero (Japan MITSUBISHI GAS CHEMICAL CO INC.) and medium CA-B from the Beijing BeNa Culture Collection.

Both bacterial strains were cultured at a temperature of 37°C and under 5% CO_2 conditions.

Animals

A total of seventy specific-pathogen-free male Wistar rats, aged 7 weeks and weighing between 210 to 240 g, were obtained from Beijing Vital River Laboratory Animal Technology Co., Ltd. (Beijing, China). The rats were housed in the Experimental Animal Center with five rats per cage, following a 12-hour light/dark cycle in a controlled environment at a constant temperature of 23°C ±2°C and a relative humidity of 55% ±5%. Throughout the study period, the rats had ad libitum access to both water and food for a duration of 7 days. All groups were provided with the same diet and water to mitigate any potential impact of diet on gut microbiota.

Animal study design

At the end of the experiments, fecal samples were collected and preserved in 1.5ml sterile centrifuge

tubes for metagenomic subsequent identification and metabolites testing. The rats were anesthetized using Isoflurane inhalation for cardiac function assessment (ECG, UCG) using an ultrasound cardiograph (VINNO 6 VET, VINNO, Suzhou China). The blood samples were collected by removal of the unilateral eyeball and preserved in 1.5ml sterile centrifuge tubes. Serum samples were isolated by centrifuges at 5000 rpm/min for 15 min at 4℃. From serum samples of five rats per group, 0.25ml of serum was extracted for biochemical testing, including Creatine Kinase isoenzymes (CKMB) and Cardiac troponin I (CTNI).

The fecal and serum samples were stored at -80℃. The heart from each rat was extracted and weighed. The hearts from five rats in each group were fixed in buffered paraformaldehyde solution (4%) for histological studies, and the other hearts were stored at -80℃. The fixed hearts were embedded in paraffin and sectioned into 3-5μm thick slices which were stained with hematoxylin and eosin (Xuebang Technology Co., Ltd., Beijing, China). All histological results were analyzed with SildeViewer 2.5 software (3D Histech, Hungary). Differences in CKMB, CTNI, wall thickness, ejection fraction (EF%) and fractional shortening (FS%) between the NA and the HH group were analyzed by the Wilcoxon rank-sum test.

Serum and fecal metabolite testing

Enzyme-linked immunosorbent assay-sandwich technique (ELISA) kits (Beijing Chengzhikewei Biotechnology Co., Ltd) were utilized to quantify the concentrations of α-KG, betaine, L-Asp, and lactate in serum samples. The samples, standards, and HRP-labelled detection antibodies were sequentially added to pre-coated microtiter wells containing the respective antibodies for α-KG, betaine, L-Asp or LAC. Subsequently, thorough washing and warming procedures were conducted.

The development of color was facilitated using the substrate TMB, which undergoes a catalytic conversion to a blue product by peroxidase and eventually turns yellow upon acidification. The intensity of the resulting yellow color is directly proportional to the concentration of α-KG in the sample. The absorbance (OD) was measured at a wavelength of 450nm using an enzyme marker, and the sample concentrations were calculated accordingly. The same process was followed for the detection of betaine and L-Asp, with the only variation being the use of specific antibodies pre-encapsulated in the microwells. Lactate ELISA kit detection range: 0.31 ng/mL-20 ng/mL; α-ketoglutarate ELISA kit detection range: 0.25ng/mL-8ng/mL; L-Aspartic acid ELISA kit detection range: 0.625 μmol/L-20 μmol/L; Betaine ELISA kit detection range: 12.5 ng/mL-400 ng/mL.

Concentrations of three metabolites (α-KG, betaine, and L-Asp) were determined in serum of rats in NA, HH, HH +*V.rogosae*, and HH +*S.rubneri* groups and the concentration of lactate was determined in serum of rats among all groups. The Wilcoxon rank-sum test was performed to evaluate the differences between all other groups and the HH group.

Rat gut microbiome analysis

To investigate whether the same variations observed in the intestinal tract of the human population were present in the rats, we collected 1.0g of feces samples per rat from both the control and model groups. All sequencing methods and raw data processing methods are consistent with the above metagenomic sequencing.

Cell culture

In this study, human AC16 cardiomyocytes (ATCC, Bethesda, MD, USA) were cultured in Dulbecco's Modified Eagle Medium/Nutrient Mixture F-12 (DMEM/F12) supplemented with 10% fetal bovine serum (Thermo Fisher Scientific) at a temperature of 37°C and under a 5% CO_2 atmosphere. The culture media were refreshed every 2 days to maintain optimal cell growth conditions.

Glycolysis analysis

The measurements were performed using a Bioscience XF96 Extracellular Flux Analyzer. Briefly, 8×10^3 cells were seeded into 96-well plates (with 4 experimental groups and 12 replicate wells per group) and incubated overnight. Subsequently, the cells were cultured with different drugs (α-Ketoglutaric acid disodium salt, L-Aspartic acid Mg salt, and betaine) under hypoxic conditions for 24 hours.

After the incubation period, the cells were washed with Seahorse buffer, and then inhibitors of the mitochondrial electron transport chain (Rot/AA) and 2-deoxy-D-glucose (2-DG) were automatically injected. Basal glycolysis was represented by the rate of glycolysis before the addition of Rot/AA, and compensatory glycolysis was represented by the rate of glycolysis in the middle segment between the addition of Rot/AA and 2-DG.

Differences in rate of glycolysis between groups were assessed by the Wilcoxon rank-sum test between all other groups and the control group.

Table S1 Demographic and clinical characteristics of PL and HA groups

Variable		PL (n=67)	HA (n=163)
Age (years)		22 (21,23)	24 (22,26)
Altitude (m)		400	4070
Residence time in Tibet (years)		-*	5 (3,7)
BMI (kg/m^2)		20.05 (19.37,20.76)	21.55 (20.62,22.86)
UCG			
	Left ventricle hypertrophy	0 (0%)	7 (4.29%)
ECG			
	Right ventricle hypertrophy	0 (0%)	3 (1.84%)
	Right axis deviation	0 (0%)	13 (7.98%)
	Incomplete right bundle branch block	0 (0%)	8 (4.91%)
FCG			
	Insufficient perfusion to myocardium	0 (0%)	21 (12.88%)
	Myocardial injury	0 (0%)	16 (9.82%)
CKMB (U/L)		11.3 (9.5,12.9)	16.0 (12.0,24.0)
CTNI (μg/L)		5.44 (3.81,6.44)	5.78 (4.88,6.75)
HBDH (U/L)		125 (108,151)	259 (204,357)
LDH (U/L)		145 (126,173)	235 (195,329)

Values are numbers (percentages) or medians (interquartile ranges). *, PL group did not migrate to Tibet.

Table S2 Demographic and clinical characteristics of HH-N and HH-A groups.

Variable	HH-N (n=42)	HH-A (n=35)
Age (years)	25.0 (22.8,26.0)	23.0 (22.0,25.0)
Altitude (m)	4070	4070
Residence time in Tibet (years)	5.00 (3.75,7.25)	5.00 (3.00,7.00)
BMI (kg/m^2)	21.19 (20.59,22.63)	21.30 (19.59,22.77)
UCG		
Left ventricle hypertrophy	0 (0%)	1 (2.86%)
ECG		
Right ventricle hypertrophy	0 (0%)	0 (0%)
Right axis deviation	0 (0%)	1 (2.86%)
Incomplete right bundle branch block	0 (0%)	1 (2.86%)
FCG		
Insufficient perfusion to myocardium	0 (0%)	13 (37.14%)
Myocardial injury	0 (0%)	12 (34.29%)
CKMB (U/L)	10 (8,11)	29 (27,37)
CTNI (μg/L)	6.05 (5.05,6.81)	5.84 (4.89,6.79)
HBDH (U/L)	192 (163,222)	357 (294,538)
LDH (U/L)	187 (160,203)	320 (263,488)
Staple food		
Rice	36 (85.71%)	30 (85.71%)
Mantou	5 (11.90%)	5 (14.29%)
Congee	1 (2.38%)	0 (0%)
Non-staple food		
Yak meat	42 (100%)	35 (100%)
Vegetable		
Seven times a week	3 (7.14%)	3 (8.57%)
Five times a week	5 (11.90%)	4 (11.43%)
Three times a week	30 (71.43%)	25 (71.43%)
One times a week	4 (9.52%)	3 (8.57%)

Values are numbers (percentages) or medians (interquartile ranges).

Table S3 Demographic and clinical characteristics of validated cohort.

Variable	Normal (n=21)	Abnormal (n=20)
Age (years)	24.0 (21.5,25.5)	24.0 (22.0,27.5)
Altitude (m)	4070	4070
Residence time in Tibet (years)	5.00 (3.50,5.00)	5.00 (2.25,7.75)
BMI (kg/m^2)	21.80 (20.16,24.17)	22.53 (21.27,23.25)
FCG		
Insufficient perfusion to myocardium	0 (0%)	20 (100%)
CKMB (U/L)	15 (14,16)	26 (24,28)
CTNI (μg/L)	5.73 (4.83,6.96)	5.43 (4.59,6.59)
HBDH (U/L)	244 (214,305)	305 (233,485)
LDH (U/L)	218 (195,278)	282 (211,423)
Staple food		
Rice	16 (76.19%)	15 (75.00%)
Mantou	4 (19.05%)	4 (20.00%)
Congee	1 (4.76%)	1 (5.00%)
Non-staple food		
Yak meat	21 (100%)	20 (100%)
Vegetable		
Seven times a week	0 (0%)	0 (0%)
Five times a week	2 (9.52%)	2 (10.00%)
Three times a week	19 (90.48%)	18 (90.00%)
One times a week	0 (0%)	0 (0%)

Values are numbers (percentages) or medians (interquartile ranges).

Table S4 369 species contributing to the differences in gut microbiota composition between the HA and PL groups

Species	LDA	P
s__Actinomycessp.ICM47	2.232608	3.09E-11
s__Bifidobacteriumadolescentis	3.002811	0.000247
s__Bifidobacteriumcatenulatum	2.158827	2.34E-05
s__Bifidobacteriumlongum	3.266646	1.76E-05
s__Collinsellaaerofaciens	2.839262	5.8E-07
s__Collinsellasp.AF39-11AT	2.003432	1.56E-05
s__Collinsellasp.AM34-10	2.122032	1.44E-07
s__Adlercreutziaequolifaciens	2.051226	1.12E-11
s__Asaccharobactercelatus	2.037172	3.34E-12
s__Eggerthellalenta	2.471944	1.75E-20
s__Eggerthellasp.AM16-19	2.006983	1.18E-07
s__Bacteroidiabacterium	2.033555	1.01E-08
s__Bacteroidalesbacterium43_36	2.120824	8.13E-11
s__Bacteroidesacidifaciens	2.130912	2.12E-08
s__Bacteroidescaccae	3.446709	1.92E-07
s__BacteroidescaccaeCAG:21	2.063874	0.000142
s__Bacteroidescellulosilyticus	2.818766	9.56E-10
s__Bacteroidesclarus	2.6256	2.4E-09
s__Bacteroidescoprocola	3.527877	2.95E-07
s__BacteroidescoprocolaCAG:162	3.020812	1.16E-05
s__Bacteroidesdorei	3.686882	5.73E-11
s__BacteroidesdoreiCAG:222	3.000858	1.29E-08
s__Bacteroidesfaecis	2.87109	1.85E-08
s__Bacteroidesfinegoldii	2.965054	1.78E-08
s__Bacteroidesintestinalis	2.836501	1.4E-11
s__Bacteroidesmassiliensis	2.970264	6.28E-07
s__Bacteroidesmediterraneensis	2.068372	5.69E-06
s__Bacteroidesnordii	2.030337	7.6E-07
s__Bacteroidesovatus	3.823226	4.97E-09
s__BacteroidesovatusCAG:22	2.720602	5.25E-08
s__Bacteroidesplebeius	4.320827	6.49E-07
s__BacteroidesplebeiusCAG:211	3.112614	7.82E-06
s__Bacteroidessalyersiae	2.621277	1.08E-09
s__Bacteroidessartorii	2.475949	3.48E-11
s__Bacteroidessp.	4.18732	7.88E-12
s__Bacteroidessp.1_1_14	2.102561	5.65E-08
s__Bacteroidessp.1_1_30	2.299996	6.57E-07
s__Bacteroidessp.2_1_22	2.554909	1.55E-08
s__Bacteroidessp.2_1_33B	2.087634	1.45E-12
s__Bacteroidessp.2_2_4	2.437185	5.93E-08
s__Bacteroidessp.3_1_13	2.245583	7.9E-06

s__Bacteroidessp.3_1_19	2.303072	1.33E-13
s__Bacteroidessp.3_1_23	2.254201	1.85E-06
s__Bacteroidessp.3_1_33FAA	2.943491	9.22E-13
s__Bacteroidessp.3_1_40A	3.056148	2.09E-08
s__Bacteroidessp.4_1_36	2.705976	2.79E-08
s__Bacteroidessp.4_3_47FAA	2.959532	4.21E-08
s__Bacteroidessp.43_108	2.35311	2.64E-13
s__Bacteroidessp.9_1_42FAA	2.565888	4.67E-11
s__Bacteroidessp.AF14-46	2.081807	2.01E-11
s__Bacteroidessp.AF25-18	2.155552	5.59E-09
s__Bacteroidessp.AF25-38AC	2.222511	1.92E-07
s__Bacteroidessp.AF29-11	2.044798	4.21E-08
s__Bacteroidessp.AF32-15BH	2.018671	2.59E-10
s__Bacteroidessp.AM16-15	2.729197	1.27E-06
s__Bacteroidessp.AM22-3LB	3.047959	0.00013
s__Bacteroidessp.AM25-34	3.340913	1.76E-06
s__Bacteroidessp.AM26-11	2.043856	4.52E-07
s__Bacteroidessp.AM32-11AC	2.013208	5.85E-12
s__Bacteroidessp.AM37-9	2.243622	7.11E-05
s__Bacteroidessp.AM44-19	2.060657	6.33E-10
s__Bacteroidessp.AR29	2.370099	3.01E-09
s__Bacteroidessp.CAG:443	2.376313	6.98E-05
s__Bacteroidessp.CAG:875	2.022376	2.77E-13
s__Bacteroidessp.CAG:98	3.194842	7.04E-05
s__Bacteroidessp.CF01-10NS	2.068192	2.94E-05
s__Bacteroidessp.D1	2.311051	4.86E-05
s__Bacteroidessp.D20	2.750436	6.08E-08
s__Bacteroidessp.D22	2.312892	0.000112
s__Bacteroidessp.KCTC15687	2.0831	1.98E-10
s__Bacteroidesstercoris	4.059162	4.76E-08
s__BacteroidesstercorisCAG:120	2.554672	1.12E-06
s__Bacteroidesthetaiotaomicron	3.476857	2.87E-11
s__Bacteroidesuniformis	3.952421	9.26E-11
s__BacteroidesuniformisCAG:3	2.401407	3.86E-08
s__Bacteroidesvulgatus	4.445029	5.07E-10
s__BacteroidesvulgatusCAG:6	2.777732	7.21E-08
s__Bacteroidesxylanisolvens	3.579752	4.37E-07
s__unculturedBacteroidessp.	3.193859	9.98E-13
s__Butyricimonasvirosa	2.078983	4.64E-05
s__Odoribactersplanchnicus	2.63455	1.3E-05
s__Paraprevotellaclara	3.125057	1.71E-09
s__ParaprevotellaclaraCAG:116	2.601248	0.001948
s__Paraprevotellaxylaniphila	2.194891	2.63E-09
s__Prevotellabivia	2.103047	4.31E-08

s__Prevotellacopri	4.441855	0.001361
s__Prevotellasp.MGM2	2.45249	7.1E-07
s__AlistipesfinegoldiiCAG:68	2.042284	2.52E-05
s__Parabacteroidesdistasonis	3.547493	2.92E-15
s__Parabacteroidesjohnsonii	2.319096	4.28E-13
s__Parabacteroidesmerdae	3.550481	6.31E-14
s__ParabacteroidesmerdaeCAG:48	2.505388	6.93E-13
s__Parabacteroidessp.20_3	2.652855	1.82E-14
s__Parabacteroidessp.AF19-14	2.277237	3.14E-15
s__Parabacteroidessp.AM44-16	2.318493	4.29E-10
s__Parabacteroidessp.CAG:2	2.809138	6.6E-13
s__Parabacteroidessp.CT06	2.052866	9.18E-15
s__Parabacteroidessp.D13	2.304548	8.23E-14
s__Parabacteroidessp.D26	2.019487	4.72E-13
s__Tannerellasp.AF04-6	2.710306	1.17E-10
s__Empedobacterbrevis	2.089894	8.72E-15
s__Firmicutesbacterium	2.585642	3.74E-05
s__FirmicutesbacteriumAF16-15	2.287093	1.46E-05
s__FirmicutesbacteriumAF36-19BH	2.542934	1.94E-05
s__FirmicutesbacteriumAF36-3BH	3.049718	1.21E-09
s__FirmicutesbacteriumAM31-12AC	2.062801	1.37E-12
s__FirmicutesbacteriumAM41-5BH	2.296574	3.7E-14
s__FirmicutesbacteriumAM43-11BH	2.082751	1.36E-14
s__FirmicutesbacteriumAM55-24TS	2.667183	4.06E-10
s__FirmicutesbacteriumCAG:110	2.309556	4.07E-05
s__FirmicutesbacteriumCAG:114	2.236765	2.67E-06
s__FirmicutesbacteriumCAG:227	2.378924	1.77E-07
s__FirmicutesbacteriumCAG:341	2.92799	0.001919
s__FirmicutesbacteriumCAG:41	2.702804	2.15E-09
s__FirmicutesbacteriumOM04-13BH	2.285651	3.23E-11
s__FirmicutesbacteriumOM07-11	2.269772	9.39E-14
s__FirmicutesbacteriumTM09-10	2.5936	0.000474
s__Lactobacillusruminis	2.008241	3.41E-08
s__Lactococcuslactis	2.224157	4.15E-05
s__Streptococcusparasanguinis	2.770788	6.86E-14
s__Streptococcussalivarius	3.282538	1.22E-09
s__StreptococcussalivariusCAG:79	2.085524	2.18E-09
s__Streptococcusthermophilus	2.280548	3.13E-08
s__Clostridialesbacterium41_21_two_genomes	2.084421	3.39E-12
s__Clostridialesbacterium42_27	2.331464	0.000129
s__Clostridialesbacterium52_15	2.646439	3.17E-07
s__Clostridialesbacterium59_14	2.684293	1.21E-06
s__ClostridialesbacteriumMarseille-P2846	2.361731	0.001116
s__Evtepiagabavorous	2.34853	5.36E-07

s__Clostridiaceaebacterium	2.616102	9.67E-11
s__Butyricicoccussp.AF10-3	2.091286	5.19E-15
s__Butyricicoccussp.AF15-40	2.433975	3.12E-14
s__Butyricicoccussp.AM05-1	2.02355	4.44E-14
s__Butyricicoccussp.AM27-36	2.099356	5.91E-10
s__Butyricicoccussp.AM28-25	2.087099	9.3E-06
s__Butyricicoccussp.OF10-2	2.059883	5.9E-14
s__Butyricicoccussp.TM10-16AC	2.002307	7.73E-15
s__unculturedButyricicoccussp.	2.368065	1.49E-13
s__Clostridiumdisporicum	2.033808	3.19E-15
s__Clostridiumsp.	2.508163	1.59E-07
s__Clostridiumsp.26_22	2.28166	7.75E-05
s__Clostridiumsp.CAG:217	2.782387	7.44E-05
s__Clostridiumsp.CAG:221	2.375993	5.9E-13
s__Clostridiumsp.CAG:265	2.335098	1.27E-14
s__Clostridiumsp.CAG:269	2.669931	0.000166
s__Clostridiumsp.CAG:433	2.516059	8.83E-09
s__Clostridiumsp.CAG:451	2.182003	5.06E-08
s__Clostridiumsp.CAG:594	2.354498	0.004231
s__Clostridiumsp.CAG:793	2.083072	0.003427
s__Clostridiumsp.SS2/1	2.581018	5.65E-11
s__unculturedClostridiumsp.	3.506268	2.73E-12
s__Hungatellahathewayi	2.931043	5.43E-10
s__Eubacteriumramulus	2.905088	1.38E-14
s__Eubacteriumsp.AM28-29	2.690512	1.27E-06
s__Eubacteriumsp.CAG:146	2.219197	2.85E-17
s__Eubacteriumsp.CAG:180	3.277146	0.000332
s__Eubacteriumsp.CAG:251	3.173857	0.000494
s__Eubacteriumsp.CAG:252	3.100516	0.000104
s__Eubacteriumsp.CAG:76	2.521263	0.002681
s__Eubacteriumsp.CAG76_36_125	2.075511	0.000792
s__Eubacteriumsp.OF10-16	2.101531	3.48E-09
s__Eubacteriumsp.TM06-47	2.003654	0.001836
s__unculturedEubacteriumsp.	2.948187	1.58E-19
s__Lachnospiraceaebacterium5_1_63FAA	2.512986	7.78E-11
s__LachnospiraceaebacteriumAM10-38	2.465549	2.97E-09
s__LachnospiraceaebacteriumAM21-21	2.344181	1.69E-10
s__LachnospiraceaebacteriumAM23-7LB	2.126137	2.89E-09
s__LachnospiraceaebacteriumAM25-27	2.176073	5.25E-11
s__LachnospiraceaebacteriumAM26-1LB	2.402295	1.22E-09
s__LachnospiraceaebacteriumOM02-26	2.45108	1.88E-09
s__LachnospiraceaebacteriumTF10-8AT	2.246556	1.15E-11
s__LachnospiraceaebacteriumTM07-2AC	2.01086	7.67E-06
s__Anaerobutyricumhallii	3.184439	8.3E-19

s__Anaerostipeshadrus	3.336541	1.02E-10
s__[Ruminococcus]gnavus	3.61522	1.05E-06
s__[Ruminococcus]torques	2.896921	2.83E-11
s__Blautiahansenii	2.22429	1.53E-14
s__Blautialuti	2.278872	1.64E-19
s__Blautiamassiliensis	2.395174	2.45E-19
s__Blautiaobeum	3.41216	1.01E-17
s__Blautiasp.	2.329175	1.64E-18
s__Blautiasp.AF14-40	2.197009	1.61E-08
s__Blautiasp.AF17-9LB	2.14072	1.76E-09
s__Blautiasp.AF19-1	2.022286	6.97E-09
s__Blautiasp.AF19-10LB	2.125334	1.92E-15
s__Blautiasp.AF19-13LB	2.097117	2.3E-09
s__Blautiasp.AF26-2	2.183256	4.47E-08
s__Blautiasp.AF32-4BH	2.331309	3.33E-08
s__Blautiasp.AF34-10	2.086117	8.47E-09
s__Blautiasp.AM28-10	2.170475	1.91E-09
s__Blautiasp.AM29-29	2.136894	1.51E-11
s__Blautiasp.AM46-3MH	2.042468	1.03E-08
s__Blautiasp.AM47-4	2.052371	6.07E-10
s__Blautiasp.BIOML-A1	2.021585	2.68E-20
s__Blautiasp.CAG:237	2.497476	5.1E-11
s__Blautiasp.KGMB01111	2.070837	2E-13
s__Blautiasp.KLE1732	2.544393	1.46E-18
s__Blautiasp.OF03-13	2.276958	8.04E-09
s__Blautiasp.OF09-25XD	2.078539	8.85E-08
s__Blautiasp.OF11-22	2.017836	8.38E-12
s__Blautiasp.OM05-6	2.24348	2.4E-11
s__Blautiasp.OM06-15AC	2.131052	3.53E-10
s__Blautiasp.OM07-19	2.359498	6.08E-12
s__Blautiasp.SC05B48	2.169717	1.03E-18
s__Blautiasp.SF-50	2.131863	2.35E-21
s__Blautiasp.TF10-30	2.011279	5.91E-10
s__Blautiasp.TM10-2	2.059398	2.26E-12
s__Blautiawexlerae	3.434615	1.22E-19
s__unculturedBlautiasp.	3.088007	8.3E-19
s__Coprococcuscatus	2.815607	1.04E-11
s__Coprococcuscomes	3.427136	2.63E-11
s__Coprococcuseutactus	2.490466	5.25E-08
s__Coprococcussp.AF27-8	2.106808	0.000137
s__unculturedCoprococcussp.	2.553814	5.11E-13
s__Doreaformicigenerans	3.336843	2.61E-18
s__Dorealongicatena	3.630837	1.97E-19
s__Doreasp.BIOML-A1	2.094632	4.71E-16

s__Fusicatenibactersaccharivorans	2.717823	9.26E-11
s__[Clostridium]aldenense	2.204481	0.000498
s__[Clostridium]bolteae	2.976085	1.14E-05
s__[Clostridium]clostridioforme	2.588966	2.67E-09
s__[Clostridium]symbiosum	2.399212	0.004174
s__Mediterraneibactersp.gm002	2.512386	1.02E-10
s__Roseburiahominis	2.808358	0.002624
s__Roseburiasp.CAG:471	2.038042	4.63E-16
s__Roseburiasp.OM02-15	2.494951	6.3E-08
s__Tyzzerellanexilis	2.52976	1.13E-09
s__Oscillibactersp.57_20	2.752012	0.000117
s__Clostridioidesdifficile	2.965615	2.1E-15
s__Intestinibacterbartlettii	2.394694	3.25E-13
s__Paeniclostridiumsordellii	2.03867	1.99E-08
s__Romboutsiailealis	2.410206	4.35E-13
s__Romboutsiatimonensis	3.288263	3.14E-12
s__Ruminococcaceaebacterium	4.134914	4.56E-05
s__RuminococcaceaebacteriumAF10-16	2.543699	0.00429
s__RuminococcaceaebacteriumTF06-43	2.399584	0.001836
s__Agathobaculumbutyriciproducens	2.300945	8.42E-15
s__Anaerotruncuscolihominis	2.233083	5.18E-06
s__unculturedAnaerotruncussp.	2.249477	9.88E-09
s__Faecalibacteriumprausnitzii	4.37684	1.38E-06
s__Faecalibacteriumsp.	3.4034	2.06E-05
s__Faecalibacteriumsp.AF10-46	2.839208	2.3E-06
s__Faecalibacteriumsp.AF27-11BH	2.860517	4.43E-05
s__Faecalibacteriumsp.AF28-13AC	3.194101	2.07E-08
s__Faecalibacteriumsp.AM43-5AT	2.791134	3.23E-06
s__Faecalibacteriumsp.BIOML-A1	2.808617	1.66E-05
s__Faecalibacteriumsp.BIOML-A2	2.393287	0.00054
s__Faecalibacteriumsp.BIOML-A3	2.894131	4.64E-05
s__Faecalibacteriumsp.CAG:82	2.007217	7.65E-05
s__Faecalibacteriumsp.OF03-6AC	2.732347	1.13E-05
s__Faecalibacteriumsp.OF04-11AC	2.417635	2.82E-07
s__Faecalibacteriumsp.OM04-11BH	2.548014	2.16E-07
s__unculturedFaecalibacteriumsp.	3.104265	7.51E-07
s__Gemmigerformicilis	3.865694	3.5E-08
s__Ruminococcusbromii	3.631015	5E-05
s__Ruminococcuscallidus	2.643877	0.000518
s__Ruminococcusfaecis	2.174551	1E-14
s__Ruminococcuslactaris	2.551494	5.82E-10
s__Ruminococcussp.	3.486841	9.96E-10
s__Ruminococcussp.5_1_39BFAA	2.064996	1.78E-19
s__Ruminococcussp.AF12-5	2.460731	4.59E-14

s__Ruminococcussp.AF14-5	2.191312	2.51E-18
s__Ruminococcussp.AF16-40	2.520677	3.67E-05
s__Ruminococcussp.AF17-11	2.330848	0.001108
s__Ruminococcussp.AF17-12	2.065418	3.53E-16
s__Ruminococcussp.AF19-29	2.000828	2.77E-18
s__Ruminococcussp.AF20-12LB	2.539194	1.37E-17
s__Ruminococcussp.AF21-42	2.008669	4.01E-19
s__Ruminococcussp.AF24-16	2.301111	1.5E-12
s__Ruminococcussp.AF24-32LB	2.003824	5.34E-14
s__Ruminococcussp.AF25-13	2.179197	7.09E-15
s__Ruminococcussp.AF25-17	2.280611	1.21E-14
s__Ruminococcussp.AF25-28AC	2.184902	9.05E-18
s__Ruminococcussp.AF27-11AA	2.291038	4.76E-15
s__Ruminococcussp.AF27-12AA	2.279426	4.72E-13
s__Ruminococcussp.AF27-3	2.221826	1.32E-11
s__Ruminococcussp.AF31-14BH	2.504565	1.02E-10
s__Ruminococcussp.AF31-16BH	2.076636	1.35E-13
s__Ruminococcussp.AF32-2AC	2.312808	7.09E-15
s__Ruminococcussp.AF37-3AC	2.496249	0.004657
s__Ruminococcussp.AF41-9	2.158928	3.25E-17
s__Ruminococcussp.AF42-10	2.193894	0.001513
s__Ruminococcussp.AF42-9BH	2.020811	2.06E-12
s__Ruminococcussp.AF43-11	2.360638	5.44E-05
s__Ruminococcussp.AF45-4BH	2.161455	2.7E-19
s__Ruminococcussp.AM09-18-1	2.112084	2.19E-14
s__Ruminococcussp.AM16-34	2.376388	3.95E-14
s__Ruminococcussp.AM22-14LB	2.386984	3.63E-14
s__Ruminococcussp.AM26-12LB	2.006638	1.02E-20
s__Ruminococcussp.AM27-11LB	2.418938	1.7E-15
s__Ruminococcussp.AM27-16	2.196055	6.43E-19
s__Ruminococcussp.AM28-29LB	2.287045	1.32E-05
s__Ruminococcussp.AM28-41	2.046057	6.82E-13
s__Ruminococcussp.AM29-12LB	2.155422	1.78E-19
s__Ruminococcussp.AM30-15AC	2.172304	1.51E-10
s__Ruminococcussp.AM31-32	2.36886	1.02E-05
s__Ruminococcussp.AM32-17LB	2.089919	1.82E-20
s__Ruminococcussp.AM33-14	2.398418	6.5E-15
s__Ruminococcussp.AM34-10LB	2.041309	0.000486
s__Ruminococcussp.AM34-9LB	2.048892	9.55E-14
s__Ruminococcussp.AM36-17	2.150336	1.42E-21
s__Ruminococcussp.AM36-18	2.339293	0.000979
s__Ruminococcussp.AM40-10AC	2.338464	2.65E-19
s__Ruminococcussp.AM41-2AC	2.283857	2.26E-11
s__Ruminococcussp.AM42-10AC	2.045235	7.06E-21

s__Ruminococcussp.AM42-11	2.517343	2.84E-16
s__Ruminococcussp.AM44-9AT	2.130971	5.47E-17
s__Ruminococcussp.AM45-2	2.257539	2.81E-19
s__Ruminococcussp.AM46-18	2.213884	9.63E-16
s__Ruminococcussp.AM49-10BH	2.422286	2.78E-09
s__Ruminococcussp.AM49-8	2.317129	9.18E-12
s__Ruminococcussp.AM57-5	2.450829	4.21E-13
s__Ruminococcussp.CAG:108	2.393768	0.000124
s__Ruminococcussp.OF02-6	2.340872	5.38E-15
s__Ruminococcussp.OF05-2BH	2.454075	4.21E-13
s__Ruminococcussp.OM02-16LB	2.199632	4.79E-19
s__Ruminococcussp.OM04-4AA	2.813008	1.49E-13
s__Ruminococcussp.OM05-7	2.545348	7.68E-20
s__Ruminococcussp.OM06-36AC	2.628759	0.00318
s__Ruminococcussp.OM07-7	2.416862	5.19E-15
s__Ruminococcussp.OM08-7	2.143054	2.19E-14
s__Ruminococcussp.TF06-23	2.062577	6.96E-19
s__Ruminococcussp.TF08-4	2.343568	1.27E-14
s__Ruminococcussp.TF10-12AC	2.36908	6.19E-13
s__Ruminococcussp.TF10-6	2.223319	1.17E-12
s__Ruminococcussp.TF11-2AC	2.243166	2E-17
s__Ruminococcussp.TF12-19AC	2.182016	0.003749
s__Ruminococcussp.TM09-4	2.271041	1.07E-18
s__unculturedRuminococcussp.	3.219176	3.64E-11
s__Subdoligranulumsp.	3.852269	9.3E-10
s__Subdoligranulumsp.60_17	3.243177	2.6E-07
s__Subdoligranulumsp.APC924/74	3.410108	4.53E-06
s__Subdoligranulumsp.OF01-18	2.361483	9.4E-08
s__Subdoligranulumsp.TF05-17AC	2.087931	7.68E-07
s__Subdoligranulumvariabile	2.634615	4.4E-11
s__Erysipelotrichaceaebacterium	2.444146	0.001877
s__Absiellasp.AM54-8XD	2.017788	4.62E-06
s__Catenibacteriummitsuokai	3.23814	1.69E-07
s__Catenibacteriumsp.	2.368247	5.86E-08
s__Catenibacteriumsp.AM22-15	2.594733	3.45E-08
s__Coprobacilluscateniformis	2.071798	5.74E-07
s__Coprobacillussp.3_3_56FAA	2.26997	3.28E-12
s__Coprobacillussp.AF02-13	2.066289	9.88E-06
s__Coprobacillussp.AF37-2	2.018374	1.42E-05
s__Coprobacillussp.AM09-26	2.147639	2.7E-06
s__Coprobacillussp.AM28-15LB	2.085823	6.23E-08
s__Coprobacillussp.CAG:183	2.292346	1.17E-08
s__Coprobacillussp.TM10-10	2.408393	5.59E-05
s__[Clostridium]innocuum	2.159336	2.03E-12

s__Erysipelatoclostridiumramosum	2.83454	2.15E-14
s__Faecalibacillusintestinalis	2.035218	4.67E-06
s__Holdemanellabiformis	3.014914	3.17E-07
s__Holdemaniafiliformis	2.384412	8.68E-05
s__Holdemaniamassiliensis	2.12127	3.17E-06
s__Phascolarctobacteriumfaecium	3.435574	1.9E-07
s__Phascolarctobacteriumsp.	2.689746	2.29E-08
s__Phascolarctobacteriumsp.CAG:207	2.908549	1.93E-09
s__Megasphaerasp.BL7	2.62177	7E-07
s__Megasphaerasp.NM10	2.81708	0.000148
s__Sutterellaparvirubra	2.342245	3.59E-07
s__Sutterellasp.63_29	2.275783	0.001095
s__Sutterellasp.AM11-39	2.613374	0.001242
s__Sutterellasp.KLE1602	2.06348	0.000177
s__Sutterellawadsworthensis	2.534012	0.000682
s__Bilophilasp.4_1_30	2.560054	5.81E-12
s__Bilophilawadsworthia	2.921072	1.56E-13
s__Tolumonassp.	2.301784	5.51E-08
s__Pseudoalteromonasruthenica	2.218885	0.000976
s__Klebsiellaquasipneumoniae	2.184262	4.73E-05

Table S5 258 species contributing to the differences in gut microbiota composition between the HH-N and HH-A groups

Species	*P*-values	Difference between means
[Propionibacterium] humerusii	0.048686	-0.000327
Acrocarpospora macrocephala	0.017838	-0.000016
Brevibacterium sp. XM4083	0.030247	-0.000001
Collinsella sp. AF29-7AC	0.049355	-0.004707
Collinsella sp. AM43-1	0.048421	-0.005611
Coriobacteriales bacterium OH1046	0.046582	-0.000174
Corynebacterium propinquum	0.037485	0.000004
Eggerthella sp. CAG:1427	0.023119	0.003681
Kribbella albertanoniae	0.027846	-0.000228
Micromonospora sp. MH33	0.029395	-0.000002
Mycobacteroides chelonae	0.034085	-0.000004
Nocardia harenae	0.043913	-0.000001
Propionibacteriaceae bacterium	0.037892	0.000006
Rhodococcus pyridinivorans	0.004422	-0.000004
Senegalimassilia anaerobia	0.040068	-0.013877
Streptacidiphilus carbonis	0.032181	0.000029
Streptomyces sp. ERV7	0.042539	-0.000006
Streptomyces sp. NBRC 110468	0.027759	0.000003
Tetrasphaera jenkinsii	0.039145	-0.000007
Trueperella bernardiae	0.048189	-0.000012
uncultured Eggerthella sp.	0.035578	-0.000014
Aquiflexum sp. Z0201	0.028172	-0.000052
Bacteroidales bacterium WCE2008	0.016569	-0.000143
Bacteroides pectinophilus CAG:437	0.043917	-0.000995
Bacteroides sp. An51A	0.022281	0.000774
Bacteroides sp. CAG:1076	0.038148	0.004316
Bacteroides sp. CAG:714	0.040455	0.000950
Bacteroides sp. HF-5287	0.037986	0.000819
Bacteroidetes oral taxon 274	0.022867	-0.000012
Duncaniella sp. TLL-A3	0.031917	0.000815
Flavobacterium croceum	0.033704	0.000010
Flavobacterium granuli	0.043392	0.000113
Mucilaginibacter sp. OK268	0.028636	0.000006
Muribaculaceae bacterium	0.040526	0.002198
Muribaculaceae bacterium Isolate-004 (NCI)	0.015871	0.000540
Muribaculaceae bacterium Isolate-042 (Harlan)	0.037374	0.000527
Muribaculaceae bacterium Isolate-077 (Janvier)	0.049067	0.000131
Muribaculaceae bacterium Isolate-083 (Janvier)	0.038953	0.000185
Muribaculaceae bacterium Isolate-084 (Janvier)	0.039917	0.000377

Muribaculum sp. TLL-A4	0.034687	0.000937
Prolixibacter bellariivorans	0.020476	-0.000052
Prolixibacter sp. NT017	0.043625	-0.000017
Proteiniphilum acetatigenes	0.038747	-0.000046
Robertkochia marina	0.047235	-0.000004
Candidatus Cryosericum odellii	0.026709	0.000052
Marinimicrobia bacterium 46_43	0.030058	-0.001931
Candidatus Saccharibacteria bacterium FS15P	0.040090	-0.000118
Candidatus Saccharibacteria bacterium GW2011_GWC2_48_9	0.004437	-0.000011
Candidatus Saccharibacteria bacterium RAAC3_TM7_1	0.013882	-0.000004
Candidatus Saccharimonas sp.	0.020615	-0.004632
TM7 phylum sp. oral taxon 346	0.039306	-0.003058
TM7 phylum sp. oral taxon 348	0.009901	-0.002338
TM7 phylum sp. oral taxon 957	0.027723	-0.000109
Candidatus Shapirobacteria bacterium GW2011_GWF2_37_20	0.043025	-0.000892
Candidatus Wolfebacteria bacterium	0.035993	0.000033
Anaerolineae bacterium UTCFX3	0.043952	-0.000001
Chloroflexus islandicus	0.045539	0.000058
Calothrix sp. HK-06	0.035174	0.000020
Gloeothece verrucosa	0.033526	-0.000002
Oscillatoriales cyanobacterium JSC-12	0.043268	0.000032
Deinococcus sp. RL	0.030443	-0.000012
Alkalibacterium kapii	0.027314	-0.000012
Alteribacillus persepolensis	0.006713	-0.000008
Anaerococcus mediterraneensis	0.036208	-0.000023
Anaerotruncus massiliensis	0.039232	-0.000896
Aneurinibacillus tyrosinisolvens	0.023989	0.000057
Angelakisella massiliensis	0.031011	-0.008617
Bacillaceae bacterium G1	0.013190	-0.000013
Bacillales bacterium	0.027822	-0.000022
Bacillus paramycoides	0.045658	0.000009
Bacillus sp. FJAT-18019	0.045591	0.000097
Bacillus sp. FJAT-28573	0.033277	0.000125
Beduini massiliensis	0.027115	0.000411
Butyricicoccus porcorum	0.004261	-0.007815
Butyricicoccus pullicaecorum	0.004107	-0.029389
Butyricicoccus sp. AM42-5AC	0.044799	0.011015
Butyricicoccus sp. OM04-18BH	0.026673	0.048546
Butyrivibrio sp. AE2032	0.016515	0.000305
Butyrivibrio sp. WCD2001	0.003791	0.000223
Caldicellulosiruptor morganii	0.047743	0.000129
Candidatus Desulfosporosinus infrequens	0.038357	-0.000015
Carnobacterium sp. WN1374	0.033792	-0.000015
Clostridiales bacterium 52_15	0.029092	-0.054147

Clostridiales bacterium 59_14	0.021958	-0.071082
Clostridiales bacterium S5-A14a	0.020249	-0.000067
Clostridiales bacterium SIT11	0.030190	0.000008
Clostridium ihumii	0.037673	0.000249
Clostridium sp. 26_21	0.045215	0.022375
Clostridium sp. 26_22	0.019525	0.060293
Clostridium sp. AF37-5	0.042483	0.010087
Clostridium sp. AM49-4BH	0.019436	0.002708
Clostridium sp. ATCC 29733	0.013299	-0.006688
Clostridium sp. HMP27	0.019653	0.000136
Clostridium sp. OM05-9BH	0.024874	0.001411
Clostridium sp. OM07-9AC	0.038031	0.001387
Clostridium sp. WB02_MRS01	0.048625	0.000455
Cohnella sp.	0.017907	-0.000011
Cohnella sp. SGD-V74	0.043729	-0.000083
Dehalobacterium formicoaceticum	0.025753	-0.001509
Dendrosporobacter quercicolus	0.040299	0.000610
Desulfofundulus australicus	0.033322	-0.000025
Desulfosporosinus sp. HMP52	0.037606	-0.000047
Desulfosporosinus sp. Sb-LF	0.014621	0.000191
Dolosicoccus paucivorans	0.046666	-0.000220
Edaphobacillus lindanitolerans	0.027299	-0.000003
Enterococcus mundtii	0.042234	0.000795
Enterococcus sp. JM9B	0.036765	-0.000934
Enterococcus timonensis	0.033617	0.000010
Epulopiscium sp. Nuni2H_MBin003	0.044085	-0.000025
Erysipelotrichaceae bacterium AM07-35-1	0.040776	0.000964
Eubacterium eligens CAG:72	0.023431	0.014345
Eubacterium plexicaudatum	0.001830	0.002667
Eubacterium sp. AF22-9	0.024756	0.027182
Eubacterium sp. AM46-8	0.031948	0.021074
Eubacterium sp. AM49-13BH	0.037082	0.018065
Eubacterium ventriosum	0.041483	0.119480
Eubacterium xylanophilum	0.026802	0.000355
Exiguobacterium sp. HF60	0.043497	0.000003
Exiguobacterium sp. Leaf196	0.026999	-0.000010
Faecalibacterium sp. OF03-6AC	0.047145	0.066009
Fictibacillus sp. S7	0.035011	0.000011
Firmicutes bacterium CAG:227	0.040356	0.080394
Firmicutes bacterium CAG:534	0.044405	-0.001387
Firmicutes bacterium CAG:646	0.031789	0.001107
Firmicutes bacterium HGW-Firmicutes-10	0.049896	-0.000032
Flavonifractor sp. An100	0.045668	-0.003313
Flavonifractor sp. An135	0.034215	-0.003722

Geobacillus stearothermophilus	0.033793	-0.002594
Halanaerobium congolense	0.048790	-0.000455
Hydrogenispora ethanolica	0.016305	-0.000675
Intestinibacillus massiliensis	0.047212	-0.000733
Jeotgalibacillus alimentarius	0.017910	-0.000001
Lachnoclostridium sp. An169	0.032059	-0.002963
Lachnospira eligens	0.028142	0.719656
Lachnospiraceae bacterium 3-1	0.004037	0.001363
Lactobacillus oligofermentans	0.011666	0.000031
Lactobacillus plantarum	0.036939	-0.001402
Lactobacillus porci	0.047789	-0.000004
Lactococcus sp. S-13	0.007946	-0.000005
Lysinibacillus sp. SYSU K30002	0.021223	0.000052
Natribacillus halophilus	0.024965	0.000003
Oribacterium sp. oral taxon 108	0.040936	-0.000089
Oscillibacter sp. 1-3	0.017121	-0.005975
Oscillibacter sp. CAG:155	0.027147	-0.004277
Paenibacillus antri	0.018373	-0.000101
Paenibacillus cellulosilyticus	0.012225	-0.000100
Paenibacillus darwinianus	0.044732	-0.000070
Paenibacillus faecis	0.042883	-0.000104
Paenibacillus macerans	0.039001	-0.002990
Paenibacillus riograndensis	0.039354	-0.000132
Paenibacillus sacheonensis	0.025903	-0.000070
Paenibacillus sp. BK673	0.041154	0.000081
Paenibacillus sp. FSL H7-0326	0.029527	-0.000028
Paenibacillus sp. GM1FR	0.049359	-0.000094
Paenibacillus sp. R196	0.049258	-0.000351
Paenibacillus taihuensis	0.024436	-0.000206
Paenibacillus terrae	0.038983	-0.000110
Paenibacillus thermoaerophilus	0.027781	-0.000035
Paenibacillus thiaminolyticus	0.024713	-0.000084
Paenibacillus timonensis	0.039927	-0.000012
Paenibacillus tuaregi	0.027327	-0.001096
Paenibacillus uliginis	0.016235	-0.000152
Pelotomaculum propionicicum	0.036637	-0.000123
Provencibacterium massiliense	0.028114	0.000577
Pseudoflavonifractor sp. 524-17	0.028207	-0.001920
Pseudoflavonifractor sp. An184	0.025209	-0.004164
Psychrobacillus soli	0.012692	-0.000022
Robinsoniella sp. RHS	0.002181	0.000704
Roseburia sp. AM16-25	0.008567	0.035407
Roseburia sp. AM51-8	0.008029	0.039071
Roseburia sp. AM59-24XD	0.011651	0.005572

Roseburia sp. CAG:100	0.022995	0.002928
Ruminococcaceae bacterium D16	0.038301	-0.017975
Ruminococcus gauvreauii	0.025663	0.000946
Ruminococcus sp. JE7A12	0.032944	0.024109
Rummeliibacillus sp. TYF005	0.029036	-0.000003
Saccharibacillus sp. O23	0.038897	-0.000022
Sporosarcina globispora	0.006982	-0.000103
Sporosarcina sp. HYO08	0.018486	-0.000032
Staphylococcus epidermidis	0.025520	0.000611
Streptococcus gallolyticus	0.047512	0.001350
Streptococcus parauberis	0.023404	0.000556
Streptococcus pluranimalium	0.020120	-0.000050
Streptococcus rubneri	0.014295	0.002821
Streptococcus sp. DD04	0.039343	-0.000053
Subdoligranulum variabile	0.029902	-0.050759
Tepidibacillus fermentans	0.020058	0.000501
Thermincola potens	0.011777	-0.000065
Thermoclostridium caenicola	0.024332	-0.000661
Trichococcus sp.	0.035519	-0.000178
uncultured Clostridium sp.	0.006276	0.323335
uncultured Oscillibacter sp.	0.038984	-0.026999
Veillonella rogosae	0.017883	0.002870
Veillonella sp. oral taxon 158	0.016630	0.002547
Veillonella sp. S13053-19	0.015955	0.003021
Virgibacillus profundi	0.048075	0.000670
Fusobacterium sp. CM21	0.006248	0.006150
Leptotrichia goodfellowii	0.006421	0.000067
Oceanivirga salmonicida	0.024309	0.000485
Streptobacillus hongkongensis	0.043574	0.000031
Lentisphaerae bacterium GWF2_45_14	0.036451	-0.000049
bacterium 0.1xD8-82	0.017141	0.000900
bacterium AMD01	0.012036	-0.000002
bacterium D16-29	0.048974	-0.002925
bacterium HR11	0.034914	0.000004
bacterium J10(2018)	0.043533	0.000771
candidate division KSB1 bacterium RBG_16_48_16	0.025461	-0.000002
candidate division KSB3 bacterium	0.040669	-0.000034
candidate division TM6 bacterium GW2011_GWF2_37_49	0.046732	0.000025
candidate division TM6 bacterium GW2011_GWF2_43_17	0.048159	0.000019
uncultured bacterium Contig1477	0.045120	-0.000007
uncultured bacterium Contig1549a	0.034907	-0.000009
uncultured bacterium Contig160	0.009666	-0.000078
uncultured bacterium Contig1761	0.048436	-0.000062
uncultured bacterium Contig29	0.013207	-0.000067

Candidatus Brocadia sp. WS118	0.035629	0.000358
Acinetobacter soli	0.032444	0.000094
Arcobacter sp. CECT 8983	0.021682	0.000059
Avibacterium gallinarum	0.039859	0.000029
Azospirillum sp.	0.033648	0.001244
Campylobacter coli	0.017579	-0.009120
Campylobacter jejuni	0.024499	-0.020755
Candidatus Accumulibacter sp. BA-91	0.032369	0.000011
Candidatus Desulfovibrio trichonymphae	0.035701	0.000038
Candidatus Filomicrobium marinum	0.047685	0.000036
Cardiobacterium sp.	0.022840	-0.000007
Corallococcus sp. AB018	0.044156	0.000010
Cronobacter universalis	0.025030	-0.000090
Desulfobacteraceae bacterium Eth-SRB2	0.015137	0.000008
Desulfocurvibacter africanus	0.012526	0.000059
Desulfovibrio indonesiensis	0.023475	0.000026
Desulfovibrionaceae bacterium CG1_02_65_16	0.047446	0.000020
Epsilonproteobacteria bacterium	0.024116	0.000255
Geobacter sp. OR-1	0.031111	0.000035
Herbaspirillum sp. ST 5-3	0.042220	-0.000004
Legionella feeleii	0.030026	0.000115
Mannheimia granulomatis	0.046208	0.000004
Mesorhizobium sp. M00.F.Ca.ET.186.01.1.1	0.029689	0.000044
Necropsobacter rosorum	0.042549	0.000008
Neisseria sp. HMSC072B12	0.008910	0.000009
Pasteurella testudinis	0.014900	0.000002
Pectobacterium actinidiae	0.047326	0.000002
Pelistega sp. NLN82	0.032993	0.000002
Photobacterium damselae	0.012459	-0.000043
Photorhabdus laumondii	0.015554	0.000019
Pseudomonas xanthomarina	0.019490	-0.000021
Rodentibacter genomosp. 2	0.029986	-0.000013
Roseovarius sp. 217	0.048215	-0.000011
uncultured Desulfovibrio sp.	0.038164	0.000702
Ursidibacter maritimus	0.016290	-0.000033
Sphaerochaeta globosa	0.028727	-0.000348
Spirochaetae bacterium HGW-Spirochaetae-2	0.012550	-0.000398
Spirochaetes bacterium GWB1_66_5	0.041162	-0.000009
Candidatus Phytoplasma australiense	0.017813	0.000510
Mesoplasma coleopterae	0.049520	0.000006
Mesotoga sp. B105.6.4	0.020601	-0.000017
Thermosipho africanus	0.015801	-0.000020
Thermosipho atlanticus	0.045939	0.000013

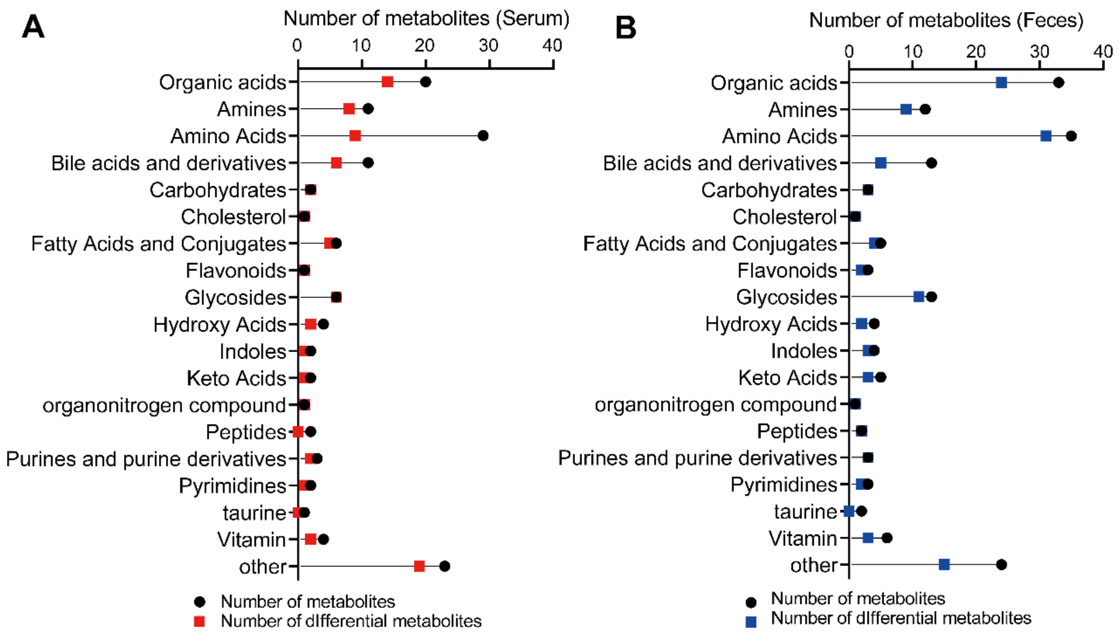

Figure S1 The number of detected metabolites and metabolites with different abundance in serum **(A)** and feces **(B)** was different between PL (n=67) and HA (n=163) groups in various metabolite classes.

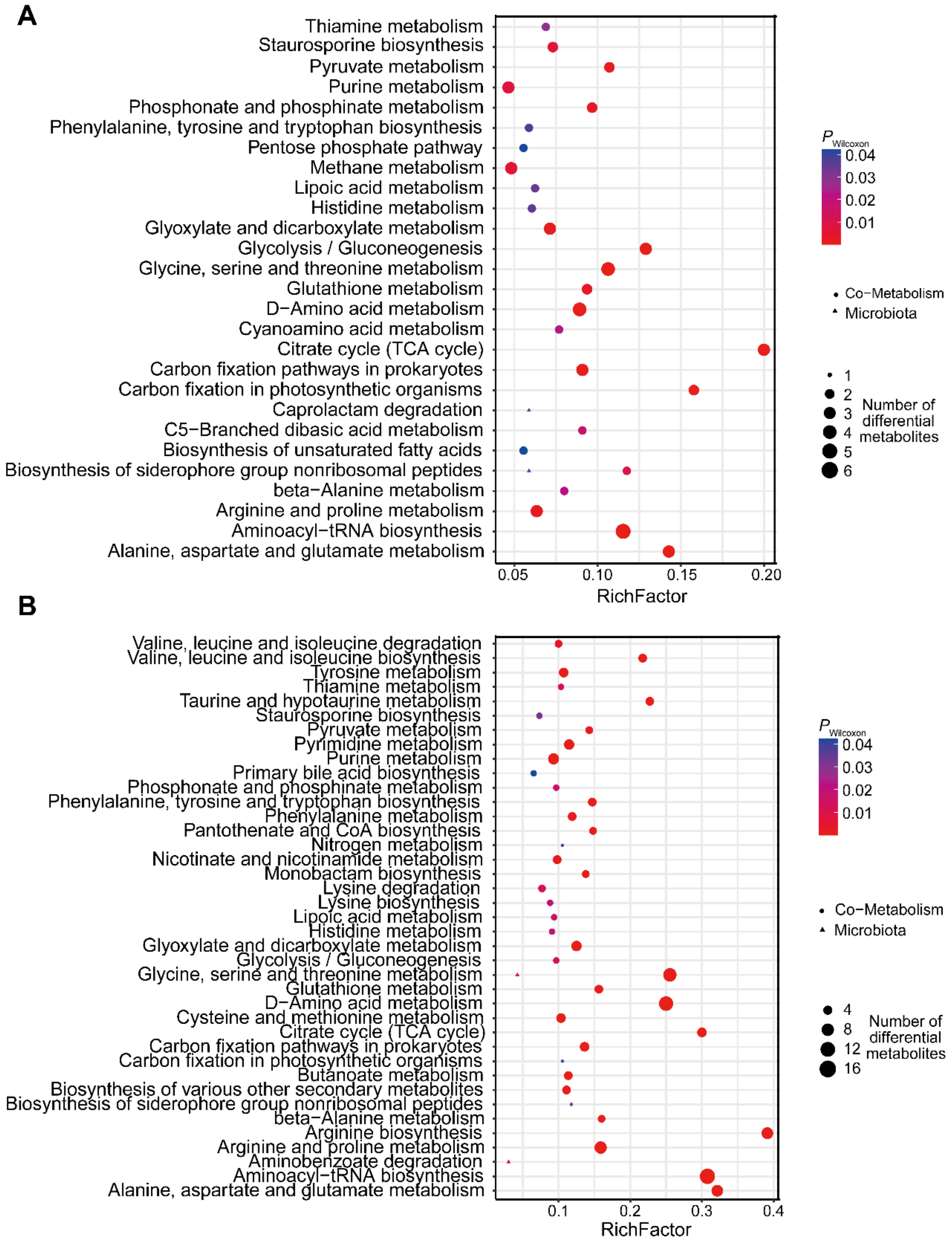

Figure S2 Enriched KEGG pathways of metabolites with different abundance in serum **(A)** and feces **(B)** between PL (n=67) and HA (n=163) groups.

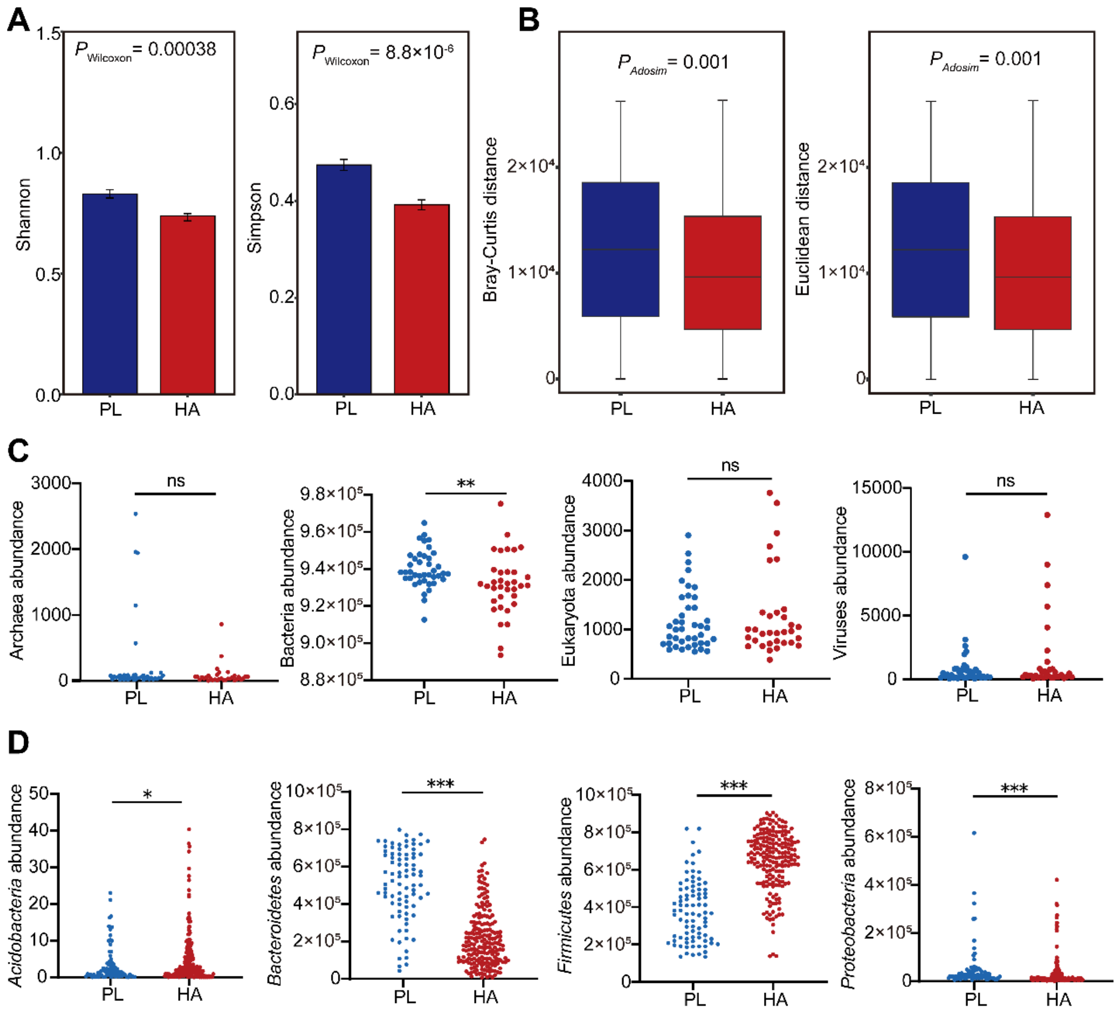

Figure S3 Differences in gut microbiota diversity between PL and HA groups. **(A)** The alpha diversity of the gut microbiota measured by Shannon and Simpson indexes in PL (n=67) and HA (n=163) groups. **(B)** The beta diversity of the gut microbiota measured by Bray-Curtis and Euclidean distance in PL (n=67) and HA (n=163) groups. **(C and D)** Differences in the abundance of gut microbiota between the PL (n=67) and HA (n=163) groups at the domain level **(C)** and the phylum level **(D)**. *, P <0.05; **, P <0.01; ***, P <0.001.

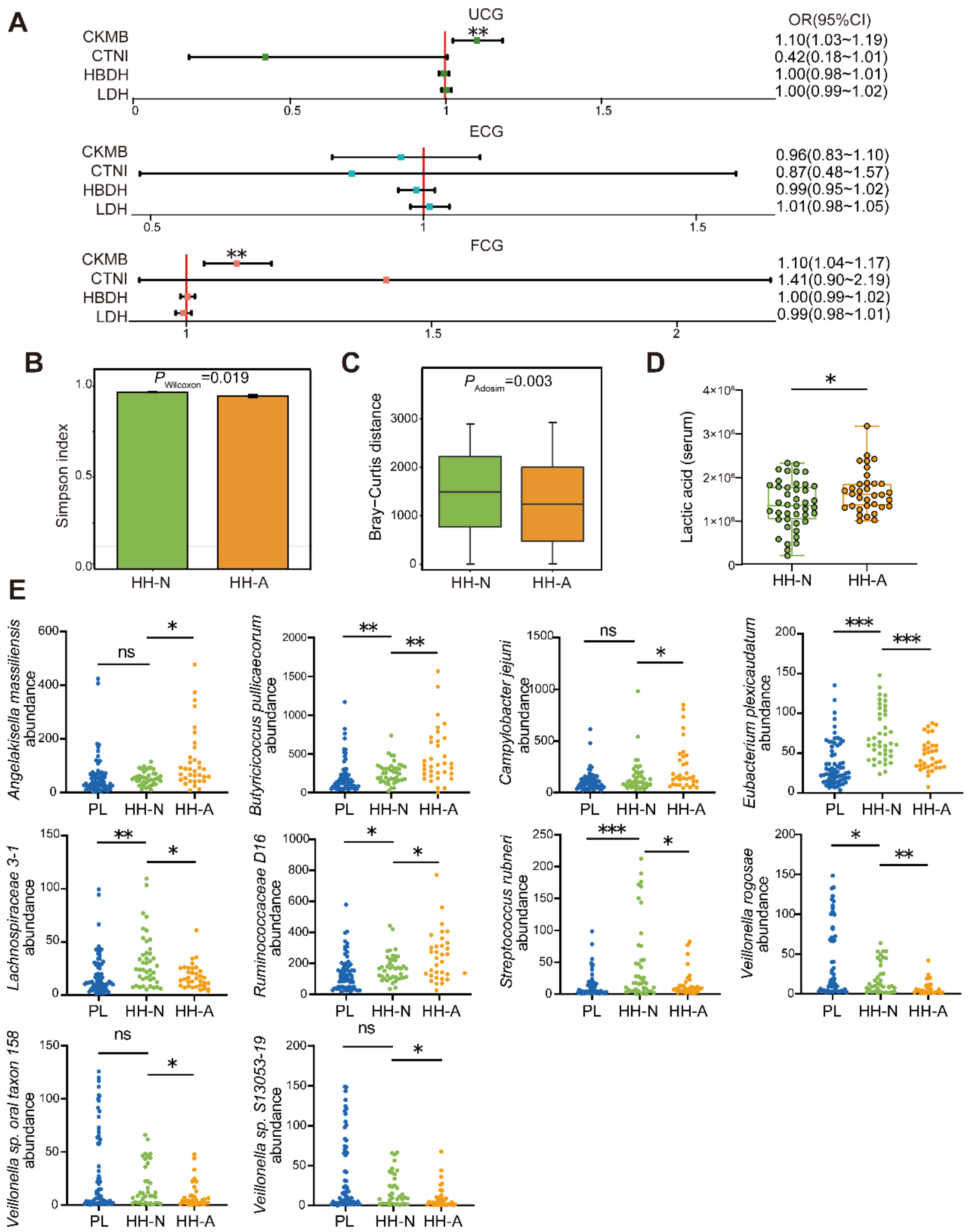

Figure S4 The association of cardiac enzymes with ECG, UCG and FCG abnormalities **(A)**. The alpha diversity of the gut microbiota measured by Simpson's index in HH-N (n=42) and HH-A (n=35) groups **(B)**. The beta diversity of the gut microbiota measured by Bray-Curtis and Euclidean distance in HH-N (n=42) and HH-A (n=35) groups **(C)**. The difference in the expression level of lactic acid between the HH-N group (n=42), HH-A group (n=35) **(D)**. The abundance differences of ten screened species among PL (n=67), HH-N (n=42) and HH-A (n=35) groups. **(E)**. *, *P* <0.05; **, *P* <0.01; ***, *P* <0.001.

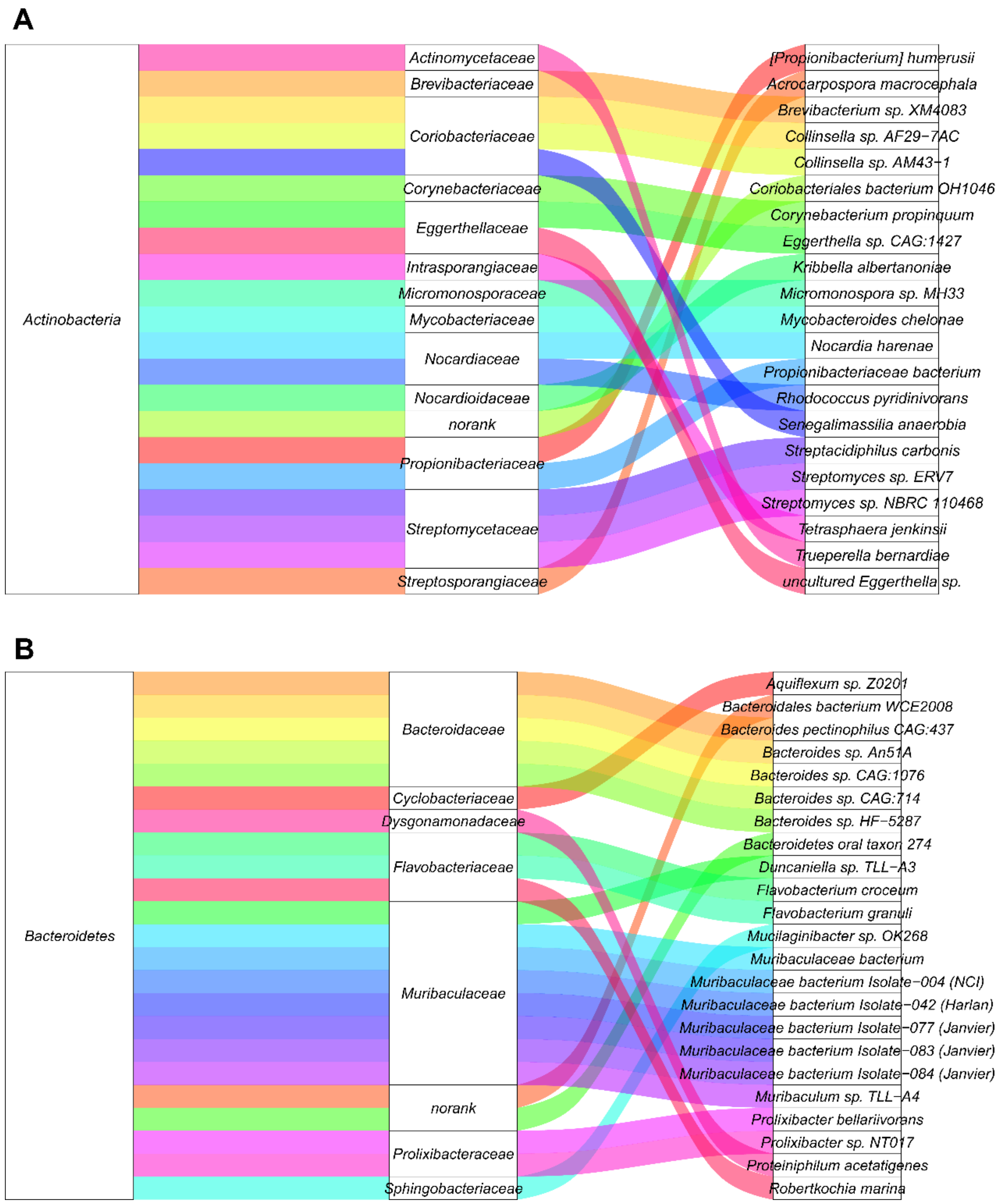

Figure S5 Classification of 258 differential species in *Actinobacteria* **(A)** and *Bacteroidetes* **(B)**.

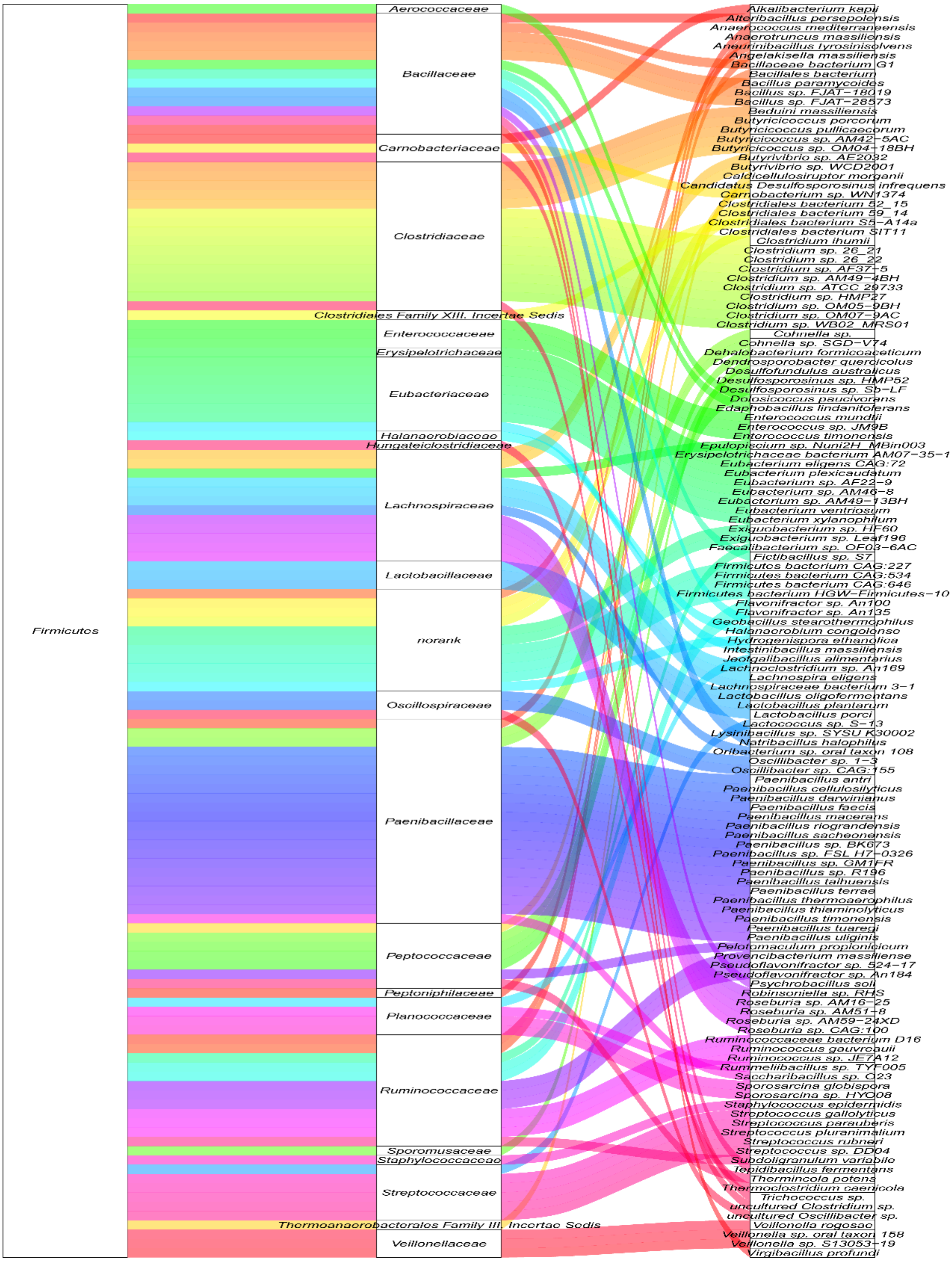

Figure S6 Classification of 258 differential species in *Firmicutes*.

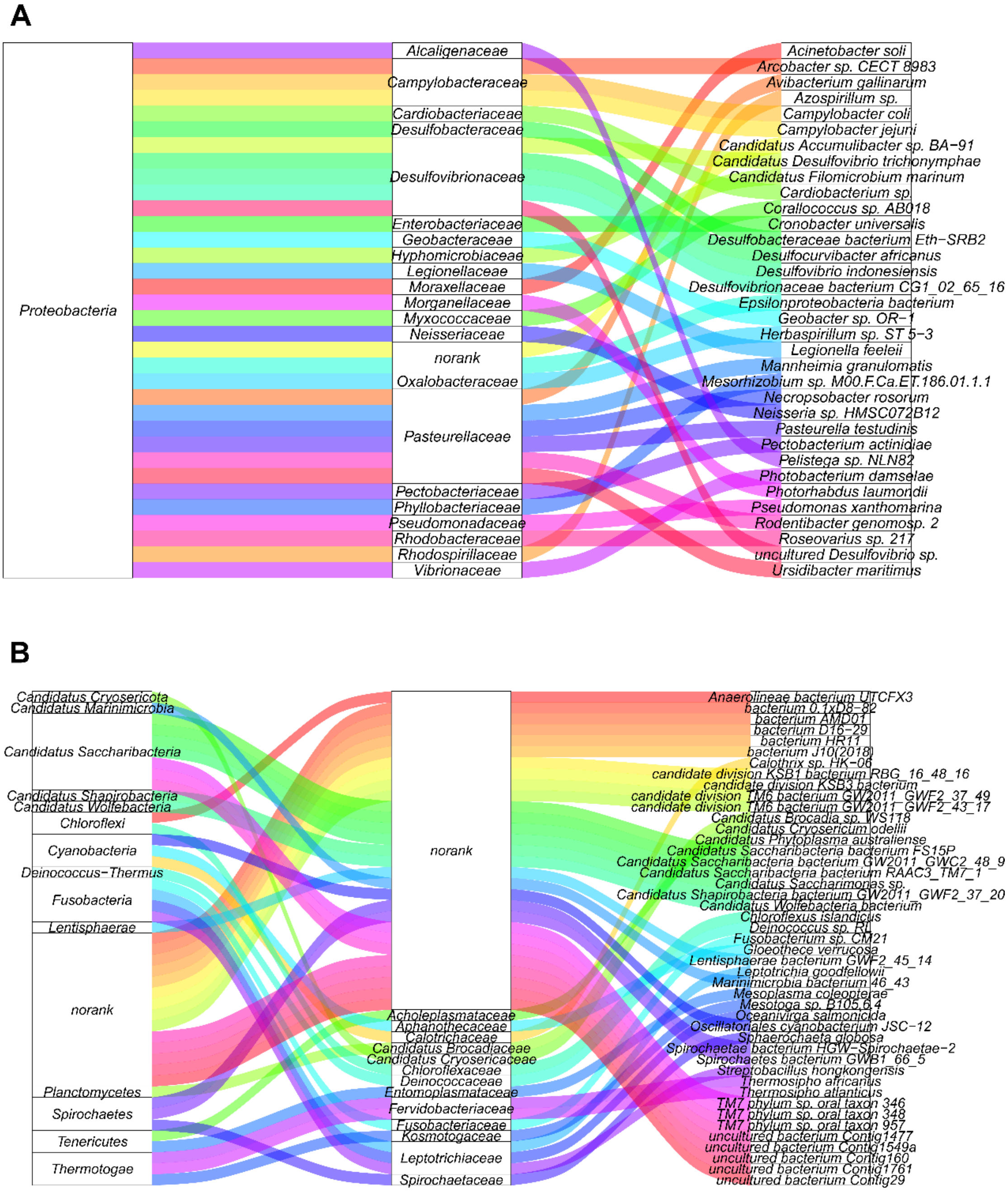

Figure S7 Classification of 258 differential species in *Proteobacteria* **(A)** and the other phylum **(B)**.

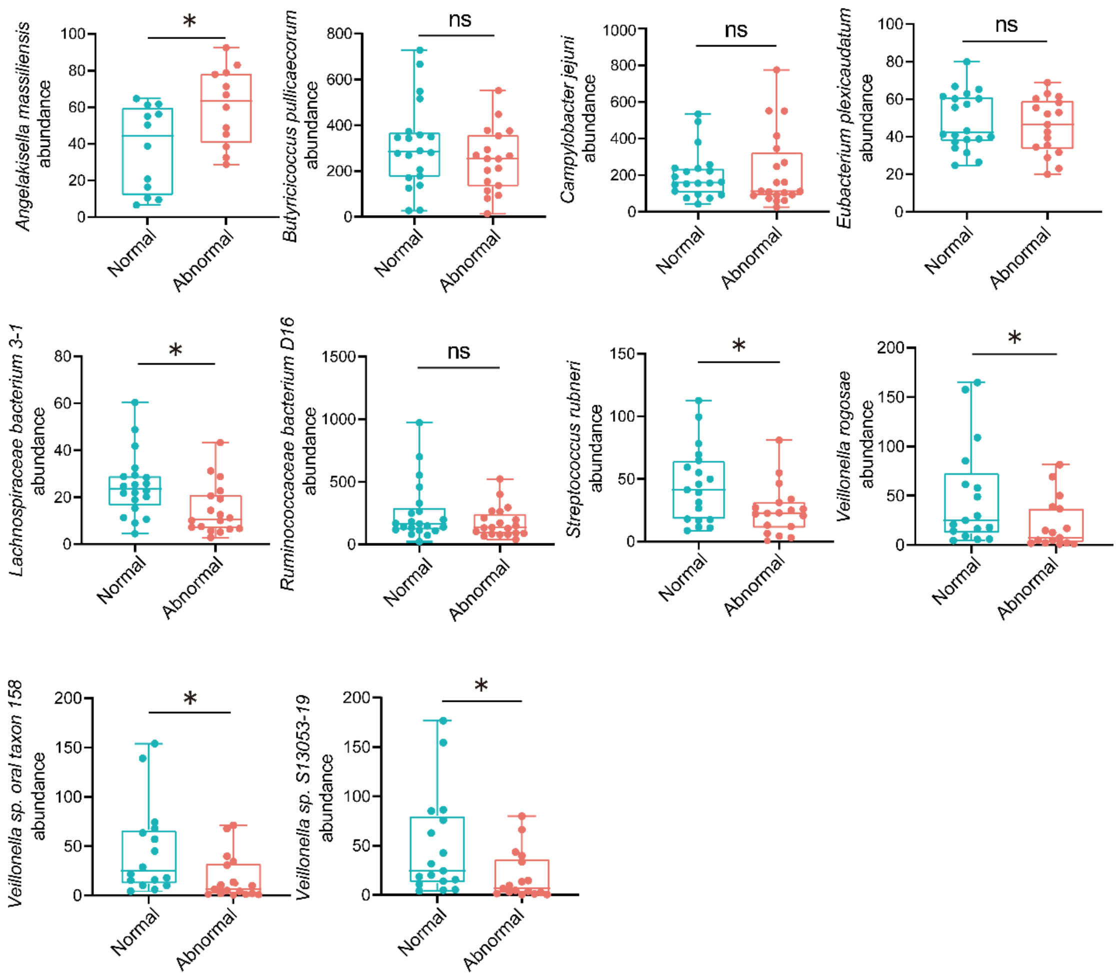

Figure S8 The abundance differences of ten screened species in validated cohort. *, *P* <0.05; **, *P* <0.01; ***, *P* <0.001.

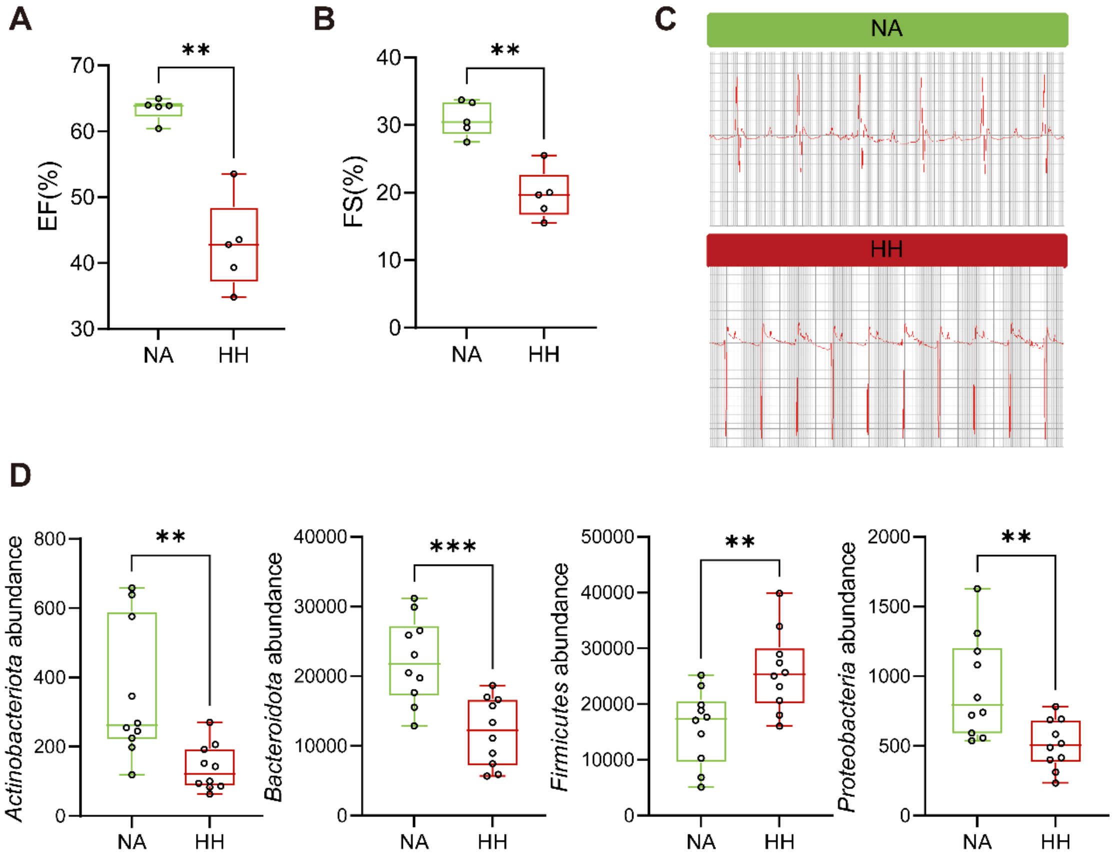

Figure S9 The differences of UCG, ECG results and four phylum abundance between NA and HH groups. **(A and B)** Ejection Fractions (EF) **(A)** and Fraction Shorting (FS) **(B)** were differed between NA (n=5) and HH (n=5) groups. **(C)** The differences in ECG results between NA and HH groups. **(D)** The abundance differences of gut microbiota at the phylum level between NA (n=10) and HH (n=10) groups. *, *P* <0.05; **, *P* <0.01; ***, *P* <0.001.

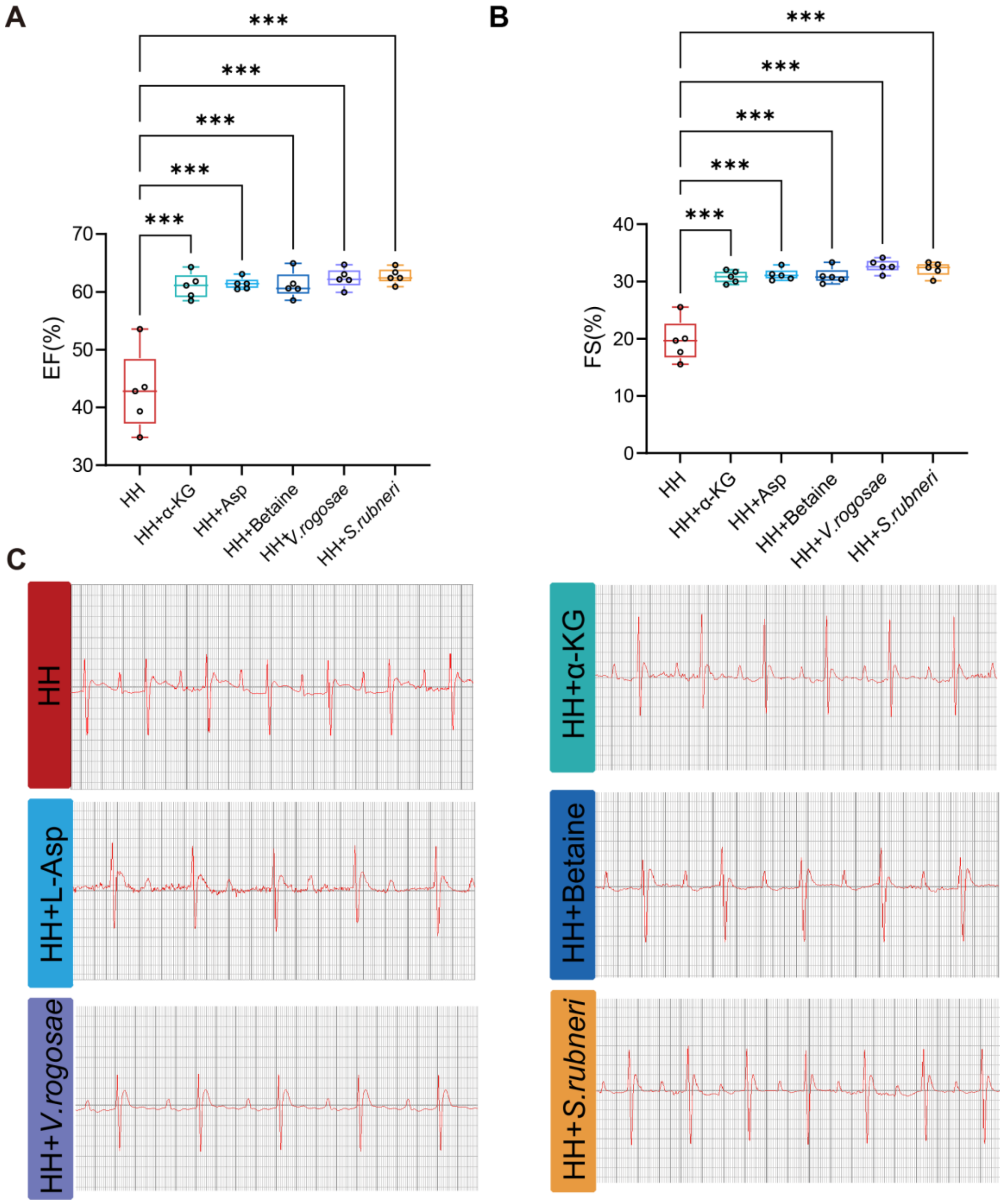

Figure S10 The differences of UCG, ECG results among HH group and intervention groups. **(A and B)** EF **(A)** and FS **(B)** were differed among HH group and intervention groups (The sample size per group is 10.). **(C)** The differences in ECG results among HH group and intervention groups. *, *P* <0.05; **, *P* <0.01; ***, *P* <0.001.
